# Multiple regulators constrain the abundance of *Caenorhabditis elegans* DLK-1 in ciliated sensory neurons

**DOI:** 10.1093/g3journal/jkaf004

**Published:** 2025-01-24

**Authors:** Yue Sun, Junxiang Zhou, Arunima Debnath, Bokun Xie, Zhiping Wang, Yishi Jin

**Affiliations:** Department of Neurobiology, School of Biological Sciences, University of California San Diego, La Jolla, CA 92093, USA; Department of Neurobiology, School of Biological Sciences, University of California San Diego, La Jolla, CA 92093, USA; Department of Neurobiology, School of Biological Sciences, University of California San Diego, La Jolla, CA 92093, USA; Department of Neurobiology, School of Biological Sciences, University of California San Diego, La Jolla, CA 92093, USA; Department of Neurobiology, School of Biological Sciences, University of California San Diego, La Jolla, CA 92093, USA; Department of Neurobiology, School of Biological Sciences, University of California San Diego, La Jolla, CA 92093, USA; Kavli Institute for Brain and Mind, University of California San Diego, La Jolla, CA 92093, USA

**Keywords:** intraflagellar transport, BBSome, MAPK-15, HSP-90, ODR-1

## Abstract

The conserved MAP3K DLKs are widely known for their functions in synapse formation, axonal regeneration and degeneration, and neuronal survival, notably under traumatic injury and chronic disease conditions. In contrast, their roles in other neuronal compartments are much less explored. Through an unbiased forward genetic screening in *C. elegans* for altered patterns of GFP-tagged DLK-1 expressed from the endogenous locus, we have recently uncovered a mechanism by which the abundance of DLK-1 is tightly regulated by intraflagellar transport in ciliated sensory neurons. Here, we report additional mutants identified from the genetic screen. Most mutants exhibit increased accumulation of GFP::DLK-1 in sensory endings, and the levels of misaccumulated GFP::DLK-1 are exacerbated by loss of function in *cebp-1*, the b-Zip transcription factor acting downstream of DLK-1. We identify several new mutations in genes encoding proteins functioning in intraflagellar transport and cilia assembly, in components of BBSome, MAPK-15, and DYF-5 kinases. We report a novel mutation in the chaperone HSP90 that causes misaccumulation of GFP::DLK-1 and up-regulation of CEBP-1 selectively in ciliated sensory neurons. We also find that the guanylate cyclase ODR-1 constrains GFP::DLK-1 abundance throughout cilia and dendrites of AWC neurons. Moreover, in *odr-1* mutants, AWC cilia display distorted morphology, which is ameliorated by loss of function in *dlk-1* or *cebp-1*. These data expand the landscape of DLK-1 signaling in ciliated sensory neurons and underscore a high degree of cell- and neurite- specific regulation.

## Introduction

The Dual Leucine zipper-bearing Kinases (DLKs) are evolutionarily conserved MAPKKKs that are broadly expressed in the nervous system and act upstream of the c-Jun *N*-terminal kinases (JNKs) and p38 MAPKs to induce signal transduction and cellular responses in a cell-type and stimuli-specific manner (Jin and Zheng 2019). A salient feature of DLKs is that they undergo auto-activation dependent on protein dimerization and oligomerization mediated by the leucine zipper domain (Holzman *et al*. 1994; Nihalani *et al*. 2000; Ikeda *et al*. 2001; Nakata *et al*. 2005; Collins *et al*. 2006). In most types of cells studied, DLKs are normally detected at low levels. However, upon insults, such as microtubule disruption drugs, traumatic injury, excitotoxicity, or pathological conditions, DLKs increase in abundance and auto-activate. Activation of DLKs can promote neural plasticity and axon regeneration, but can also lead to synapse disruption, cell death, and axon degeneration (Jin & Zheng 2019). Several conserved mechanisms and factors have been shown to constrain the activity of DLKs, including ubiquitin-mediated protein degradation by the PHR E3 ligases (Nakata *et al*. 2005; Collins *et al*. 2006; Lewcock *et al*. 2007), the HSP chaperone network (Daviau *et al*. 2006; Karney-Grobe *et al*. 2018; Zheng *et al*. 2020), JNK-interacting proteins (JIPs) (Nihalani 2001; Sengupta Ghosh *et al*. 2011; Kulkarni *et al*. 2021), palmitoylation enzymes (Holland *et al*. 2016; Niu *et al*. 2020), and calcium and cAMP signaling (Ghosh-Roy *et al*. 2010; Yan & Jin 2012; Hao *et al*. 2016). Emerging evidence has also implicated abnormal DLK signaling in stroke and neurodegenerative conditions (Huang *et al*. 2017; Joy *et al*. 2019). Despite the progress made, the low abundance of DLKs remains a challenge in understanding how DLKs are regulated in vivo.

Forward genetic screening in *C. elegans* is a powerful unbiased approach to uncover unsuspected genes and their functional networks with physiological relevance. Previous genetic screens for suppressors of the E3 ligase *rpm-1* mutants revealed in vivo function of DLK-1 in synapse formation and its regulation by protein degradation (Nakata *et al*. 2005), as well as at the level of mRNA processing (Noma *et al*. 2014). Other genetic screens subsequently revealed critical roles of DLK-1 in response to microtubule disruption, in axon regeneration, and axon patterning (Hammarlund *et al*. 2009; Yan *et al*. 2009; Bounoutas *et al*. 2011; Zheng *et al*. 2020). Thus far, our understanding of how the expression of DLK-1 is regulated has largely relied on cell-type transgenic manipulation, and how endogenously expressed DLK-1 is regulated remains to be examined. The advent of genome editing has enabled the insertion of fluorescent proteins to any desired genomic loci. We recently described endogenous DLK-1 expression using a GFP knock-in reporter. Combining with forward genetic screening using GFP::DLK-1, we uncovered a mechanism whereby intraflagellar transport in ciliated sensory neurons regulates the abundance of DLK-1 in cilia. Disruption of intraflagellar transport causes misaccumulation of DLK-1 in defective cilia and also increases expression of CEBP-1 in nuclei of sensory neurons, which in turn represses transcription of *dlk-1* (Sun & Jin 2023). Here, we report the characterization of additional mutants. Our analysis extends the previous conclusion that DLK-1 abundance in ciliated neurons involves a feedback regulation mediated by its downstream transcriptional factor CEBP-1. We also find that the guanylyl cyclase *odr-1* represses DLK-1 and CEBP-1 signaling in the AWC neurons, and show that *dlk-1* and *cebp-1* antagonize *odr-1* in AWC cilia morphology. Our findings reveal that ciliated neurons use multiple pathways to constrain DLK-1 abundance and expand the cellular and molecular landscape of DLK-1 signaling.

## Materials and methods

### *C. elegans* genetics

Animals were grown on nematode growth medium (NGM) plates seeded with *Escherichia coli*  OP50 as described (Brenner 1974). The wildtype strain was Bristol N2. Generation and characterization of the GFP knock-in allele *GFP::dlk-1(ju1579)* were described previously (Sun & Jin 2023). All experiments were performed with hermaphrodites; males were used for genetic crosses and for assessing chromosome linkage. Compound mutants were generated following standard procedures and verified by phenotypes and genotyping. Strains and their genotypes are in Supplementary Table 1. Information on alleles and genotyping methods is in Supplementary Table 2.

### Mutagenesis and visual screening

We mutagenized L4 animals of CZ26350  *GFP::dlk-1(ju1579); rpm-1(ju44); cebp-1(tm2807*) using 50 mM ethyl methane sulphonate following the standard procedure (Brenner 1974). After washing with M9 buffer, mutagenized P0 animals were recovered on a freshly seeded NGM plate for 1 hour at room temperature. About 60 healthy P0 animals were transferred in a group of 10 to 6 seeded NGM plates to produce F1 progenies. 3 days later, 50 F1s at L4 stage from each P0 plate were transferred, in groups of 5 or 10, to freshly seeded NGM plates for 4 consecutive days, totaling of ∼1,200 F1s on 240 plates. After culturing at room temperature for 3–4 days, about 30 F2 progeny at L4 stage from each F1 plate were mounted in M9 buffer onto microscope slides, and the pattern and intensity of GFP::DLK-1 were examined under a 63× oil objective on a Zeiss Axioplan 2 microscope equipped with a Semrock GFP/DsRed-A-ZHE filter (excitation 468/553 nm, emission 512/630 nm). Non-mutagenized L4 animals of CZ26350 were used as same-day control. Putative F2 mutants showing visually discernable altered pattern and/or intensity of GFP::DLK-1 were recovered from the microscope slides to propagate; their progenies (F3 and F4) were re-examined to confirm altered GFP::DLK-1 expression. Only 1 F2 mutant line from an F1 plate was saved to ensure independent isolates. We did not pursue any mutants that showed sterility or larval lethality. After outcrossing, we obtained 27 independent mutants that displayed visibly increased GFP::DLK-1 at the anterior tip of the animals.

### Outcrossing and assessment of X linkage

CZ25941  *GFP::dlk-1(ju1579)* was used in the initial outcrossing of each mutant line. A total of 12–16 L4 or young adult *GFP::dlk-1(ju1579)* males were transferred with OP50 to the center of an unseeded NGM plate and mated with 3–4 L4 mutant hermaphrodites. Parallel crosses of CZ25941  *GFP::dlk-1(ju1579)* were used as a negative control, while crosses of CZ25941  *GFP::dlk-1(ju1579)* males to CZ26728 *GFP::dlk-1(ju1579); cebp-1(tm2807) ifta-1(ju1644)* were used as a positive control (Sun & Jin 2023). Wild-type-looking F1 cross-progeny hermaphrodites were propagated individually to produce F2s. After 3–4 days, 20–30 L4-stage F2 progenies from each F1 plate were mounted onto a microscope slide using ∼0.2 μl M9 containing 1 mM levamisole, and re-examined under compound fluorescence microscope. Outcrossed F2 animals showing GFP::DLK-1 misaccumulation resembling the original mutants were recovered from the slide to propagate to F3 generation, which were then genotyped for *rpm-1(ju44)* and *cebp-1(tm2807*). We assessed linkage to chromosome X by examining >30 F1 male cross-progeny for GFP::DLK-1 under a compound fluorescence microscope. If F1 males, but not hermaphrodites, showed detectable misaccumulation of GFP::DLK-1 resembling the original mutants, the mutants were tentatively assigned to chromosome X.

### Whole-genome sequencing analysis and verification of candidate mutations

Genomic DNAs were prepared using a Puregene Cell and Tissue Kit (Qiagen, cat#158689). 20× coverage of whole-genome sequencing (WGS) data was obtained as 90/100/150 bp paired-end reads (BGI America). Raw reads were mapped to the *C. elegans* reference genome (WS235/ce11) using Burrows–Wheeler Aligner (BWA) in the Galaxy platform (http://usegalaxy.org) (Giardine *et al*. 2005; Li & Durbin 2009). Mutagenesis-induced nucleotide variants (SNPs) were obtained by subtracting those present in the lab N2 strain (CZ21293) using the Genome Analysis Toolkit (GATK)-based “Select Variants” tool on the Galaxy platform. To identify candidate mutations, we extracted strain-specific SNPs by comparing non-outcrossed and outcrossed strains of the same allele. We verified putative causal SNPs by Sanger sequencing on further outcrossed strains. If available, we examined previously reported independent alleles.

### DiI uptake assay

The stock solution (2 mg/ml) of DiI (1,1′-dioctadecyl-3,3,3′,3′-tetramethylindo-carbocyanine perchlorate) (Molecular Probe, cat# D-282) was made in DMSO, and used in 1:200 or 1:1,000 dilution. Briefly, mix-staged animals were washed twice with 1 ml M9 solution, resuspended in DiI-containing M9 buffer, and incubated under foil at room temperature for 1 hour on a slow shaker. After washing twice with M9, animals were transferred to a seeded NGM plate to recover for ∼30 minutes. Animals were first assessed for DiI uptake under a fluorescence dissection microscope; healthy L4 animals were then mounted on microscope slides with 4% agar pad and scored for DiI uptake in amphids and phasmids under 63× lens on a Zeiss fluorescence compound microscope. About 40–60 animals per strain in 2–3 independent assays were scored in a genotype-blind manner, along with same-day controls.

### Confocal microscopy imaging

L4 animals were anaesthetized in M9 buffer containing 1 mM levamisole on microscope slides with 10% agarose pads, and imaged using a 63× oil immersion objective on a Zeiss LSM800 confocal microscope. Laser settings were adjusted for each fluorescence marker as described below.

#### GFP::DLK-1 and CEBP-1::GFP

Images of GFP::DLK-1*(ju1579)* were acquired using EGFP channel (488 nm excitation peak), laser power at 10%, detection wavelength at 485–700 nm, detector gain at 650 V, scan speed at 2.53 s/frame. Z-stack images of the entire anterior region from the nose tip to the grinder in the terminal bulb of pharynx were acquired at 0.5 μm interval for 12–15 slices, which covered left or right side of the nerve ring and head ganglia. For quantifying GFP::DLK-1 intensity in the cilia area, Z-stack images were acquired with 0.25 μm interval for 7–10 sections, covering either the left or right side of bilateral symmetric sensory endings of ciliated neurons. Images of CEBP-1::GFP*(st12290)* were collected using EGFP channel (488 nm excitation peak), laser power at 1%, detection wavelength at 400–533 nm, detector gain at 600 V, scan speed at 1.27 s/frame. Z-stack images of the whole body from L2 animals were acquired with 0.5 μm interval for 22–30 slices for projection. Fluorescence intensity was quantified using Fiji software as described previously (Sun and Jin 2023). Briefly, the cilia regions were outlined as regions of interest, and average signal intensity were measured by subtracting background fluorescence intensity. The fluorescence intensity of each sample was normalized to the average fluorescence intensity value in the same-day control group.

#### Colocalization of GFP::DLK-1 with glia or EV markers

We imaged L4 animals that co-expressed GFP::DLK-1*(ju1579)* and the transgene *nsEx1153*, which labeled amphid sheath cells (*F16F9.3pro*-mCherry) and amphid socket cells (*itr-1pro*-CFP) (Heiman & Shaham 2009). Images were acquired in 3 different channels with following adjusted laser settings: EGFP channel (488 nm excitation peak), laser power at 0.8%, detection wavelength at 400–560 nm, detector gain at 650 V, scan speed at 2.53 s/frame; mCherry channel (561 nm excitation peak), laser power at 0.5%, detection wavelength at 570–700 nm, detector gain at 650 V, scan speed at 2.53 s/frame; CFP channel (405 nm excitation peak), laser power at 2%, detection wavelength at 400–500 nm, detector gain at 700 V, scan speed at 2.53 s/frame. When imaging GFP::DLK-1*(ju1579)* with the amphid sheath cell transgene marker *F16F9.3pro-CFP* (*juEx8158*) (Razzauti & Laurent 2021), we used the following imaging settings: EGFP channel (488 nm excitation peak), laser power at 1%, detection wavelength at 515–700 nm, detector gain at 650 V, scan speed at 10.13 s/frame; CFP channel (405 nm excitation peak), laser power at 2%, detection wavelength at 400–500 nm, detector gain at 650 V, scan speed at 10.13 s/frame. Despite optimization of imaging settings, slight bleed-through from mCherry or CFP to GFP channel was unavoidable. However, such bleed-through fluorescence showed pattern and localization distinguishable from GFP::DLK-1 and was useful for outlining amphid sheath cells in the cilia region in the merged Z-stack images. We imaged GFP::DLK-1*(ju1579)* and TSP-6::wrmScarlet(*syb4211*) (Razzauti & Laurent 2021) using GFP channel (488 nm excitation peak), laser power at 0.8%, detection wavelength at 400–560 nm, detector gain at 650 V, scan speed at 5.06 s/frame, and mCherry channel (561 nm excitation peak), laser power at 0.5%, detection wavelength at 570–700 nm, detector gain at 650 V, scan speed at 5.06 s/frame. Z-stack images were taken with 0.25 μm interval for 7–10 sections, covering either left or right side of bilateral symmetric cilia and amphid sheath cell.

### Generation of *hsp-90/daf-21* single-copy transgene

Full-length genomic DNA of *hsp-90/daf-21*, including 1,246 bp 5’ upstream and 118 bp 3′ downstream sequences flanked by LoxP sites, was cloned into pDEST5605 by Gateway cloning. The resulting plasmid was injected into EG8079  *oxTi179; unc-119(ed3)*; *juSi183[daf-21(+); cb-unc-119(+)]* was selected following the protocol as described (Frøkjær-Jensen *et al*. 2008).

### Statistical analysis

Statistical analysis was performed using GraphPad Prism 10 (GraphPad Software, Inc.). Statistical significance was determined using Fisher's exact test or Welch's ANOVA test for multiple comparison corrected with FDR method of Benjamini and Hochberg, as appropriate. *P* > 0.05 was considered not significant (ns). *P* < 0.05 (*), *P* < 0.001 (***), and *P* < 0.0001 (****) were considered significant differences. Data are represented as mean ± SEM. The numbers of individual samples are shown in columns in corresponding figures.

## Results and discussion

### Genetic screen design and overview of mutants altering the pattern and abundance of GFP::DLK-1

**Fig. 1.**
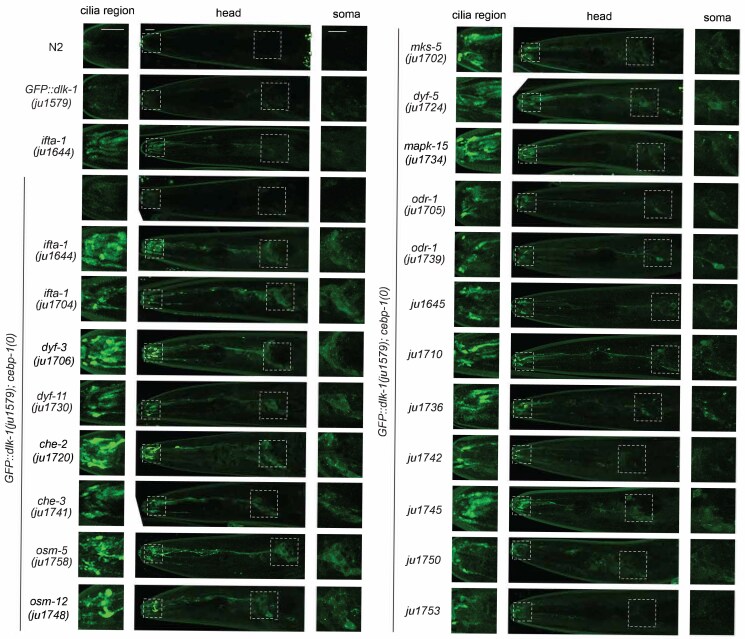
Genetic mutants showing misaccumulation of GFP::DLK-1 in ciliated neurons. Representative confocal Z-stack images in the head regions of L4 animals of indicated genotypes. Cilia regions and amphid soma are indicated by white dashed boxes and enlarged in the left and right panels. Scale bar: 5 μm.

We aimed to isolate mutants that could reveal new genes regulating the spatial distribution and abundance of DLK-1 expressed from the endogenous genomic locus. As previously described (Sun & Jin 2023), GFP was inserted in-frame with the ATG of both the long and short isoforms of DLK-1 (Yan & Jin 2012), designated GFP::DLK-1(*ju1579*). Homozygous GFP::DLK-1(*ju1579*) animals retained the normal function of DLK-1, and fluorescence was largely invisible under compound fluorescence microscopy (Fig. 1). We tested several genes reported to regulate DLK-1 expression, and found that *rpm-1(0)* mutants showed a consistent increase of GFP::DLK-1 fluorescence in the nerve ring by about 2-fold (Sun & Jin 2023). To perform the genetic screen, we chose to mutagenize *GFP::dlk-1(ju1579); rpm-1(0); cebp-1(0)* animals (CZ26350) for 2 reasons. First, the inclusion of *rpm-1(0)* provided a stable baseline for visual detection of GFP::DLK-1 and also prevented the isolation of new mutations in *rpm-1* and its known partners (Liao *et al*. 2004; Grill *et al*. 2007, 2016). Second, as overexpression of *dlk-1* is known to cause animals to be unhealthy and slow-growing (Nakata *et al*. 2005), we included *cebp-1(0)* to reduce potential toxic effects associated with *dlk-1* overexpression, which might increase the chance of isolating mutants with potential growth defects. *cebp-1(0)* did not affect GFP::DLK-1 fluorescence intensity on its own or in *rpm-1(0)* mutants (Sun & Jin 2023) (Fig. 1).

We conducted visual screening in a semi-clonal fashion as described in the *Materials and methods*. After outcrossing using non-mutagenized *GFP::dlk-1(ju1579)*, we obtained 27 independent mutants that showed increased accumulation of GFP::DLK-1 in the anterior tips of the animals and behaved as single recessive mutations. All outcrossed mutants retained *cebp-1(0)* (Fig. 1, Supplementary Table 1), consistent with our previous conclusion that *cebp-1(0)* enhanced visual detection of GFP::DLK-1 misaccumulation in sensory neurons (Sun & Jin 2023). Several mutants showed linkage to chromosome X; *ju1703* co-segregated with *rpm-1(0)*, suggestive of a linkage to chromosome V. We performed WGS analysis on both the original and outcrossed strains (Supplementary Table 1 and *Materials and methods*), and focused on mutagenesis-induced SNPs that were present in both strains. For independent mutants that contained function-disrupting SNPs in the same gene, we performed non-complementation tests between independent alleles, or with previously reported loss-of-function mutations in the respective genes, when possible. We also performed a lipophilic dye-uptake assay and found multiple mutants to be strongly defective (Table 1). Together, using the streamlined analysis, we were able to assign molecular changes to 20 mutations in 12 genes. We did not find consistent candidate mutations for 7 mutants (*ju1645*, *ju1710*, *ju1736*, *ju1742*, *ju1745*, *ju1750*, *ju1753*) (Fig. 1, Table 1), which could be due to low quality or depth of sequencing reads, or poorly annotated genes. We previously reported 7 mutations in 4 genes, *ifta-1*, *daf-10*, and *che-3*, which function in intraflagellar transport, and *che-10* that functions to maintain basal body integrity (Sun & Jin 2023). Here, we describe 13 mutations with likely molecular changes.

**Table 1.** Summary of alleles, dye-fill, and other phenotypes.

Allele	Gene (mutation)	Chromosome linkage*^a^*	Dye-fill assay*^b^*		Other associated phenotypes
			Amphids	Phasmids	
*Control^c^*			100% dye-fill	100% dye-fill	
*ju1644^d^*	ifta-1(W103*)	X	0% dye-fill	0% dye-fill	
*ju1704*	ifta-1(R822*)	X	42% partial dye-fill*^e^*	0% dye-fill	DiI intensity in filled neurons varied.
*ju1706*	*dyf-3* *(g.685G to A at 5′ splice site)*	IV	0% dye-fill*^f^*	0% dye-fill*^f^*	
*ju1730*	*dyf-11(M1I)*	X	0% dye-fill*^f^*	0% dye-fill*^f^*	
*ju1720*	*che-2* (W615*)	X	0% dye-fill*^f^*	0% dye-fill*^f^*	
*ju1741*	che-3 (W3792*)	I	2% dye-fill*^f^*	ND	
*ju1758*	*osm-5* *(g.1312G to A at 5′ splice site)*	X	0% dye-fill*^f^*	0% dye-fill*^f^*	
*ju1748*	osm-12/bbs-7 (R496*)	III	0% dye-fill*^f^*	0% dye-fill*^f^*	
*ju1702*	*mks-5* (Q431*)	II	2% dye-fill 98% partial dye-fill*^e^*	65% partial dye-fill*^e^*	
*ju1724*	*dyf-5(K6Q)*	Linked to *dlk-1 (ju1579) I*	5% dye-fill *^f^*	ND	
*ju1734*	*mapk-15* *(g.1028G to A at 5′ splice site)*	III	93% partial dye-fill*^e^*	15% partial dye-fill*^e^*	Dye-filled neurons often showedgranular pattern in cilia and dendrites.
*ju1703*	*hsp-90/daf-21* *(S564F)*	Linked to *rpm-1 V*	100% dye-fill	40% partial dye-fill*^e^*	
*ju1705*	*odr-1(S894F)*	X	ND	ND	
*ju1739*	*odr-1*(W202*)	X	100% dye-fill	100% dye-fill	
*ju1645*	*ND*	Unlikely linked to V or X	100% dye-fill	100% dye-fill	
*ju1710*	*ND*	Unlikely linked to V or X	30% dye-fill 70% partial dye-fill*^e^*	95% dye-fill 5% partial dye-fill*^e^*	Slow growing, some larvae appeared to be dauer-like. Dye-filled neurons frequently had granular appearance; some sensory neurons appeared to be mispositioned.
*ju1736*	*ND*	Unlikely linked to V or X	100% partial dye-fill*^e^*	67% partial dye-fill*^e^*	
*ju1742*	*ND*	Unlikely linked to V or X	100% dye-fill	100% dye-fill	
*ju1745*	*ND*	Unlikely linked to V or X	33% partial dye-fill*^e^*	43% partial dye-fill*^e^*	Slow growth, small brood size, some embryonic lethality. DiI intensity in filled neurons varied, some exhibiting granular appearance, low penetrance of mispositioning of sensory neurons.
*ju1750*	*ND*	Unlikely linked to V or X	5% partial dye-fill*^e^*	5% partial dye-fill*^e^*	
*ju1753*	*ND*	Unlikely linked to V or X	100% dye-fill	100% dye-fill	

^*a*^Chromosome linkage to X was based on GFP::DLK-1 misaccumulation phenotypes in F1 cross-progeny males, but not in F1 cross-progeny hermaphrodites. Linkage to V was based on co-segregation with *rpm-1(ju44) V*.

^*b*^Dye-fill assay was carried out using either 1/200 dilution or 1/1,000 dilution of 2 mg/ml DiI stock. Animals were stained for 1 hour, and scored under compound fluorescence scope using 63× lens, except those noted under e. % represents the score from >40 L4-YA animals per strain in 2–3 independent dye-fill assays using 1/1,000 DiI dilution. All strains also have *GFP::DLK-1(ju1579)* and *cebp-1(0)*.

^*c*^Controls are: CZ25941 *GFP::dlk-1(ju1579)*, *CZ27822 GFP::dlk-1(ju1579)*; *cebp-1(tm2807)*, or CZ26350 *GFP::dlk-1(ju1579); rpm-1(ju44); cebp-1(tm2807)*.

^*d*^ *ifta-1(ju1644)* was reported in Sun and Jin (2023), and used here for comparison.

^*e*^Partial dye-fill defects refer to less than 6 pairs of amphids or less than 2 pairs of phasmids showing variable DiI fluorescence in neuronal soma.

^*f*^Dye-fill assay on these alleles was done using 1/200 dilution of 2 mg/ml DiI stock, and scored under dissection scope.

### New alleles of genes functioning in intraflagellar transport and cilia formation

In control animals *GFP::DLK-1*(*ju1579)* and *GFP::DLK-1(ju1579); cebp-1(0)*, GFP fluorescence was barely visible in the cilia region, dendritic bundles and soma of amphid neurons (Fig. 1). In null mutants of *ifta-1* or *daf-10*, GFP::DLK-1 fluorescence was increased to about 2-fold in the cilia stump and distal dendrites, compared to control animals (Sun & Jin 2023). In all strains characterized, the presence of *cebp-1(0)* mutation enhanced the detection of misaccumulated GFP::DLK-1 in sensory endings, with visible fluorescence also detectable along dendritic shaft and soma of sensory neurons (Fig. 1). The enhanced effect of *cebp-1(0)* was specific to sensory neurons, as GFP::DLK-1 fluorescence intensity and pattern in the nerve ring and nerve cords were indistinguishable from the control animals. A few mutants, including *ju1736*, *ju1748*, and *ju1720*, exhibited some punctate pattern of GFP::DLK-1 in the somatic cytoplasm of sensory neurons. In DiI uptake assay, animals of *ju1706*, *ju1720*, *ju1730*, *ju1741*, *ju1748*, and *ju1758* showed strong defects, while others showed variable degrees of defects in some amphids and/or phasmids (Table 1). Two mutants, *ju1710* and *ju1745* showed slow growth and small brood size. A brief summary of the 8 mutants affecting genes in cilia transport and assembly follows.

*ifta-1(ju1704)* (CZ30404 *GFP::dlk-1(ju1579); cebp-1(tm2807) ifta-1(ju1704)*) behaved as an X-linked mutation during outcrossing analysis. It contains a C-T nucleotide transition in *ifta-1*, resulting in a stop codon at Arg822 (Table 1, Supplementary Fig. 1). IFTA-1 has multiple WD40 repeats in the N-terminus, but no annotated domain or motif after Arg822. *cebp-1(0) ifta-1(ju1704)* animals showed misaccumulation of GFP::DLK-1 in the cilia region, which was visibly weaker than that in *cebp-1(0) ifta-1(ju1644)* animals (Fig. 1). The R822* mutation may cause destabilization of mRNA or compromised protein function due to C-terminal truncation.

*che-3(ju1741)* (CZ30406 *che-3(ju1741) GFP::dlk-1(ju1579); cebp-1(tm2807)*) contains a G-A nucleotide change, resulting in a stop codon at Trp3792 in the Dynein heavy Chain CHE-3 (Table 1, Supplementary Fig. 1). CHE-3 contains multiple coiled-coil domains, an AAA domain, a stem and a stalk domain. Animals of CZ30406 showed nearly complete dye-uptake defects. Increased GFP::DLK-1 intensity in the cilia region in CZ30406 was visibly weaker than that in *che-3(ju1729); cebp-1(0)*, suggesting that *che-3(ju1741)* is a partial loss of function.

**Fig. 2.**
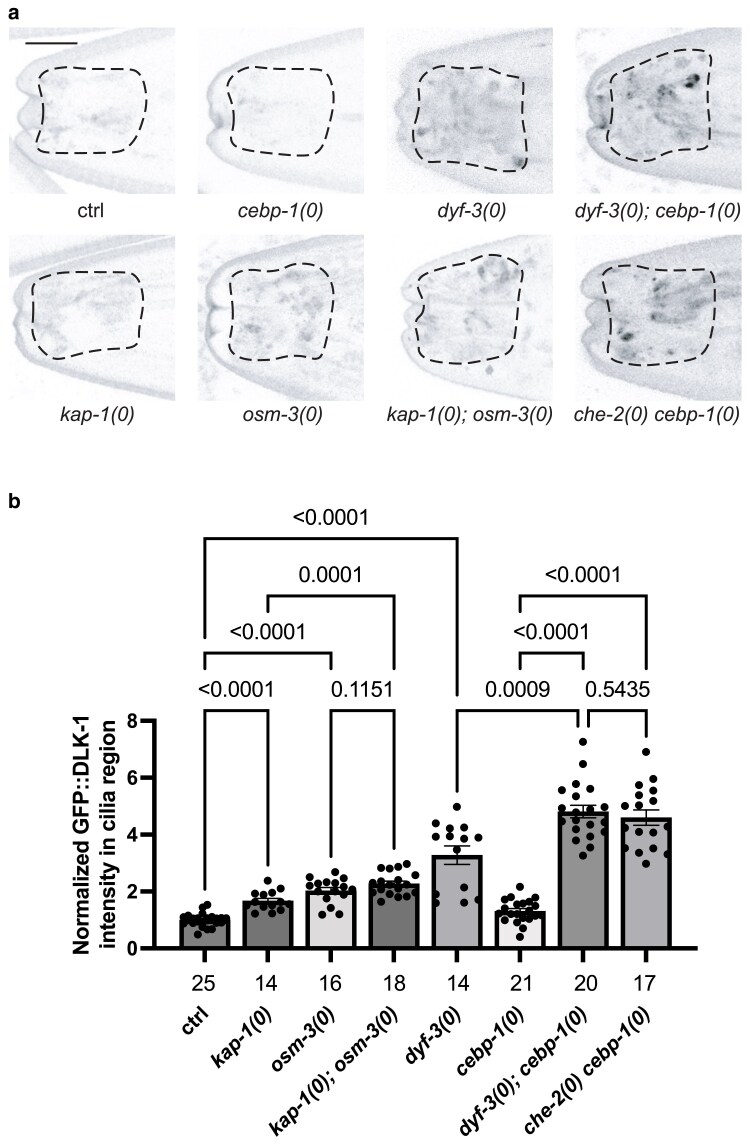
Anterograde IFT kinesins function in constraining levels of GFP::DLK-1 in sensory neuron cilia. a) Representative confocal Z-stack images and b) quantification of GFP::DLK-1 expression in the cilia region of L4 animals of indicated genotypes. Null *(0)* alleles used are *kap-1(ok676)*, *osm-3(p802)*, *dyf-3(ju1706)*, *che-2(ju1720)*, and *cebp-1(tm2807)*. Cilia regions are indicated by dashes. Scale bar: 5 μm. Data are represented as mean ± SEM, with each dot representing an individual animal; the total numbers of animals per genotype are shown below the columns. Statistics: Welch's ANOVA test with multiple comparison, Benjamini and Hochberg FDR corrected. *P*-values are shown above brackets between the compared columns.

*dyf-3(ju1706)* (CZ30233 *GFP::dlk-1(ju1579); dyf-3(ju1706); cebp-1(tm2807)*) contains a G-A nucleotide transition at the 5′ splicing donor site at exon-intron 3 junction of *dyf-3*, which encodes the ortholog of human Clusterin-associated protein 1(CLUAP1), a component of IFT-B complex (Murayama *et al*. 2005) (Table 1, Supplementary Fig. 1). Altered splicing in *ju1706* likely leads to out-of-frame after Asp182. We tested an independent allele *dyf-3(m185)*, which has a G-A substitution that results in a stop codon at Arg 163 (Murayama *et al*. 2005), and observed increased GFP::DLK-1 accumulation in the cilia area. The intensity of GFP::DLK-1 misaccumulation was further increased in *dyf-3(m185); cebp-1(0)*, compared to *dyf-3(m185)* single mutants (Fig. 2a and b). The levels of misaccumulated GFP::DLK-1 in the cilia region in CZ30233 were visibly comparable to other strong loss-of-function mutations in *ift* genes, such as CZ27185 (*GFP::dlk-1(ju1579); cebp-1(tm2807) ifta-1(ju1644)*) (Fig. 1). These data suggest that *dyf-3* functions in the IFT-dependent feedback regulation of DLK-1.

*dyf-11(ju1730)* (CZ28087 *GFP::dlk-1(ju1579); cebp-1(tm2807) dyf-11(ju1730)*) behaved as an X-linked mutation during outcrossing analysis, and has a G to A nucleotide change that alters the initiation ATG to ATA in *dyf-11* (Table 1, Supplementary Fig. 1). *dyf-11* encodes the ortholog of human TNF Receptor-Associated Factor Interacting Protein 1(TRAF3IP1)/IFT54, a subunit of IFT-B complex (Kunitomo & Iino, 2008). *dyf-11(ju1730)* likely reduces the synthesis of full-length protein. Animals of CZ28087 showed 100% dye-uptake defects (Table 1), although misaccumulated GFP::DLK-1 in the cilia region was visibly weaker than that in CZ27185  *GFP::dlk-1(ju1579); cebp-1(tm2807) ifta-1(ju1644)* (Fig. 1).

*che-2(ju1720)* (CZ30234 *GFP::dlk-1(ju1579); cebp-1(tm2807) che-2(ju1720)*) contains a G-A nucleotide change in *che-2*, resulting in a stop codon at Trp615 (Table 1, Supplementary Fig. 1). *che-2* encodes a WD40-containing protein orthologous to human IFT80, an IFT-B protein (Fujiwara *et al*. 1999). Animals of CZ30234 showed 100% dye-fill defects (Table 1) and visibly increased GFP::DLK-1 in the cilia region, sensory dendrites bundles and many amphid soma (Fig. 1).

*osm-5(ju1758)* (CZ30407 *GFP::dlk-1(ju1579); cebp-1(tm2807) osm-5(ju1758)*) showed linkage to the X chromosome, and contains a G-A mutation on the splicing site of *osm-5*, which encodes the ortholog of human IFT88, an IFT-B protein (Qin *et al*. 2001) (Table 1, Supplementary Fig. 1). Animals of CZ30234 showed 100% dye-uptake defects (Table 1) and increased GFP::DLK-1 fluorescence in the cilia region, with diffused fluorescence noticeable in a dozen or so amphid neuron soma (Fig. 1).

*mks-5(ju1702)* (CZ30403 *GFP::dlk-1(ju1579); mks-5(ju1702); cebp-1(tm2807)*) contains a C-T nucleotide change that causes a stop codon at Gln431 in MKS-5 (Table 1, Supplementary Fig. 1), the orthologue of mammalian MKS5/RPGRIP1L/NPHP8, which regulates the formation of ciliary transition zone (TZ) (Jensen *et al*. 2015; Li *et al*. 2016). Animals of CZ30403 showed misaccumulation of GFP::DLK-1 in the cilia region, with the overall intensity less than that in CZ27185  *GFP::dlk-1(ju1579); cebp-1(tm2807) ifta-1(ju1644)*) (Fig. 1).

*dyf-5(ju1724)* (CZ30398 *dyf-5(ju1724) GFP::dlk-1(ju1579); cebp-1(tm2807)*) contains an A-C nucleotide change, resulting in Lys6Gln missense mutation in the MAK kinase DYF-5 (Table 1, Supplementary Fig. 1). Lys 6 is predicted to be in a β-sheet by alpha-fold (Supplementary Fig. 2a), and is conserved among close homologs. We previously examined *dyf-5(ok1177)*, a null allele, which showed elevated expression of DLK-1 and CEBP-1 in ciliated neurons (Sun & Jin 2023). The overall intensity of GFP::DLK-1 misaccumulation in CZ30398 appeared to be less than that in *dyf-5(ok1177); cebp-1(0)*. The Lys6Gln mutation may alter kinase activity or protein localization.

In summary, the identification of the above new alleles adds further evidence that intraflagellar transport acts in concert to constrain DLK-1 abundance.

### IFT kinesins also repress the abundance of DLK-1 in ciliated sensory neurons

Intraflagellar transport is bi-directional and involves both anterograde motor kinesins and retrograde motor dynein, along with motor adaptor complexes IFT-A and IFT-B. Although our screen was not performed to a saturation level, we were able to isolate multiple mutations in a single gene (e.g. *ifta-1*, *che-3*), as well as single mutations in multiple genes that are components of IFT-A or IFT-B. As we did not find any mutation in IFT kinesin genes, we tested null alleles of *kap-1* and *osm-3*, encoding components of heterotrimeric and homodimeric kinesin-2 motors, respectively (Snow *et al*. 2004). Both *kap-1(ok676)* and *osm-3(p802)* animals showed modest and significant GFP::DLK-1 misaccumulation in the cilia region, but to a lesser degree than in other IFT-A or IFT-B mutants (Fig. 2a and b). *osm-3(p802); kap-1(ok676)* double mutants displayed GFP::DLK-1 accumulation in cilia region indistinguishable from *osm-3(p802)* single mutants. This analysis shows that ciliary kinesin II is also involved in constraining DLK-1 levels, but may likely act in parallel with other genes.

### GFP::DLK-1 in cilia is not detectable in amphid glial cells, and unlikely released as extracellular vesicles in *ift* mutants

**Fig. 3.**
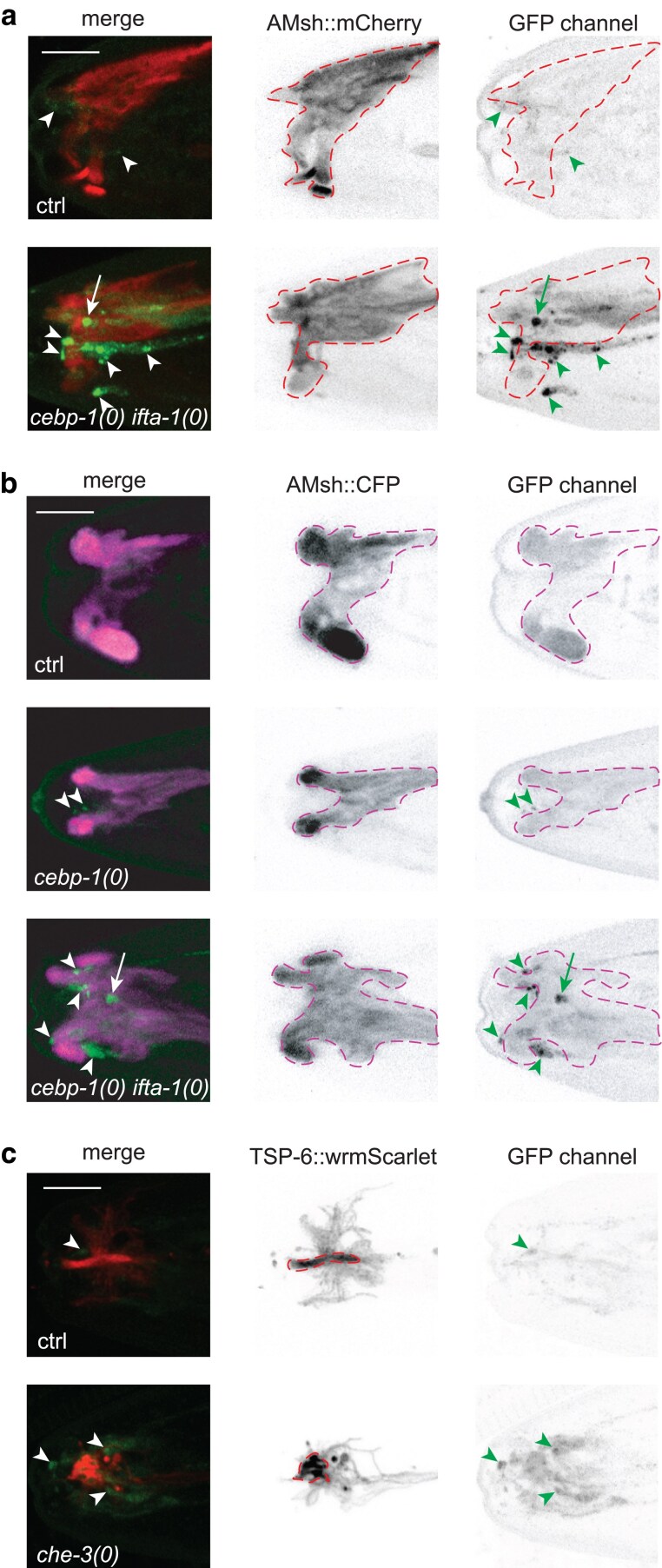
GFP::DLK-1 is undetectable in glial cells or extracellular vesicles. Shown are representative confocal Z-stack images co-expressing GFP::DLK-1 with AMsh::mCherry (a, amphid sheath cells outlined by dashes, DLK-1 accumulation outside amphid sheath cell pointed by arrowheads; DLK-1 accumulation underlying but not overlapping with the amphid sheath cell region pointed by arrows), AMsh::CFP (b, ctrl: no GFP::DLK-1*(ju1579)*, amphid sheath cells outlined by dashes, DLK-1 accumulation outside amphid sheath cell pointed by arrowheads, DLK-1 accumulation underlying but not overlapping with the amphid sheath cell region pointed by arrows), and TSP-6::wrmScarlet (c, PCMC areas outlined by dashes, DLK-1 accumulation pointed by arrowheads). Images are in merged channels (left panel) and single channels (middle and right panels). Scale bar: 5 μm.

The anterior tip where GFP::DLK-1 misaccumulated in *ift* mutants also contains amphid glial cells and surrounding epidermis. It has been reported that some sensory neurons in *ift* mutants can release extracellular vesicles (EVs), which can be taken up by amphid glial cells (Razzauti & Laurent 2021). Low abundance and diffused fluorescence of GFP::DLK-1 in control and *ift* mutants raise an open question of whether DLK-1 could also be present in glial cells and whether DLK-1 could be released in EVs. We previously showed that in multiple *ift* mutants misaccumulation of GFP::DLK-1 in cilia led to up-regulation of CEBP-1::GFP only in sensory neurons, not in amphid glial or anterior epidermal cells (Sun & Jin 2023), suggesting that DLK-1 is unlikely expressed or misaccumulated in the other cell types around the anterior tip. Here, we further examined animals co-expressing GFP::DLK-1 with amphid sheath cell markers. In control animals (*GFP::dlk-1(ju1579)* or *GFP::dlk-1(ju1579); cebp-1(0)*), fluorescence signals of GFP::DLK-1 were undetectable in amphid sheath cells. In *cebp-1(0) ifta-1(ju1644)* animals, the increased accumulation of GFP::DLK-1 around the anterior tip did not show detectable overlap with CFP or mCherry labeled amphid sheath cells (Fig. 3a and b). Additionally, to test if GFP::DLK-1 might be released in EVs, we examined colocalization of GFP::DLK-1 with TSP-6::wrmScarlet, which labels EVs released from ciliated neurons (Razzauti & Laurent 2021). In *che-3(0)*, biosynthesis and release of EVs are enhanced, leading to accumulation of TSP-6::wrmScarlet in amphid sheath cells. We observed that in *che-3(0)* mutants misaccumulated GFP::DLK-1 in cilia area did not show colocalization with TSP-6::wrmScarlet (Fig. 3c). With the caveat inherent to detection limitation on fluorescence intensity of these reporters, this analysis suggests that DLK-1 in normal or defective cilia is unlikely present in amphid glial cells and unlikely to be released in EVs.

### BBSome components may differentially affect DLK-1 in ciliated neurons

**Fig. 4.**
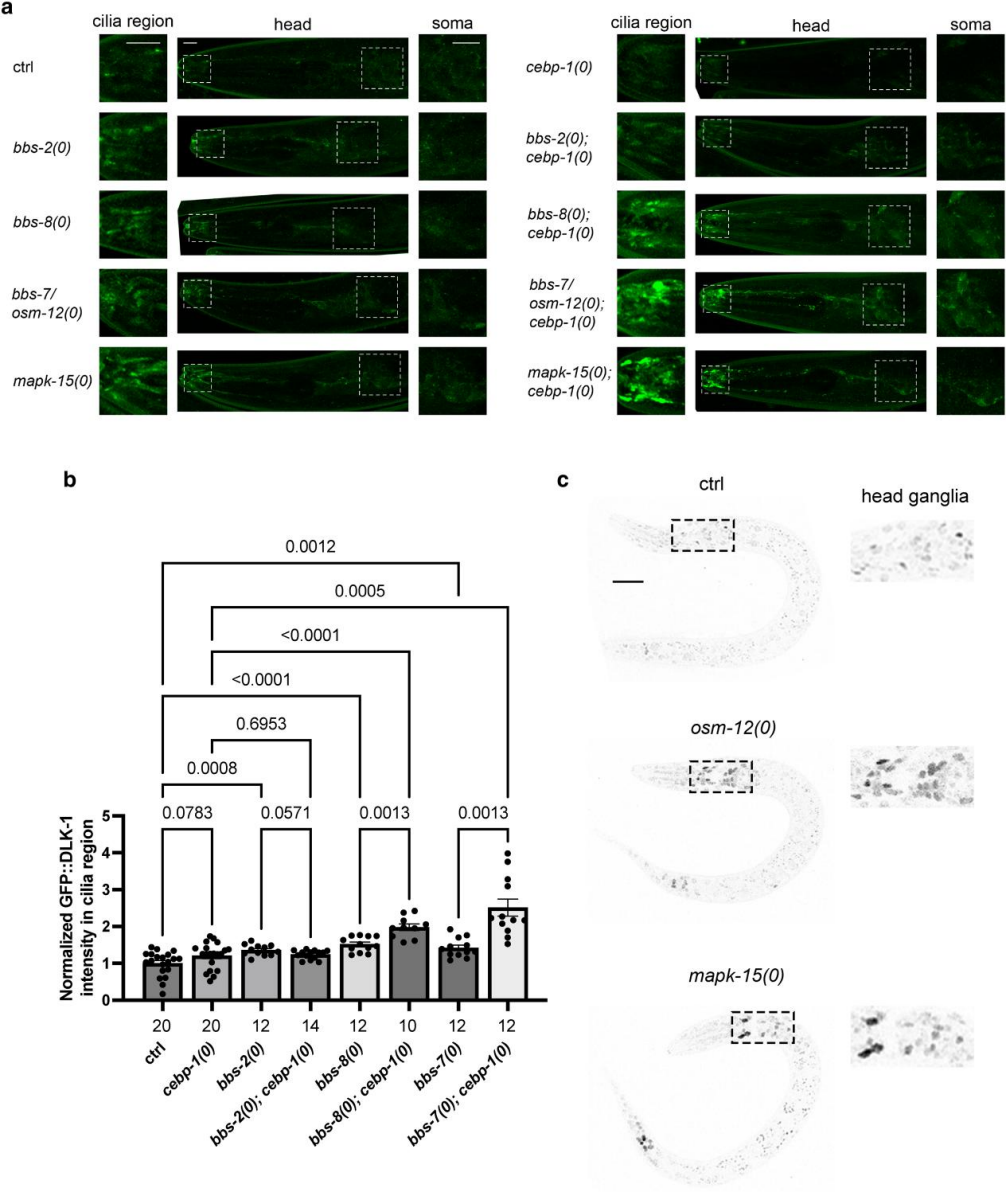
BBSome genes and *mapk-15* constrain levels of GFP::DLK-1 in ciliated neurons. a) Representative confocal Z-stack images of GFP::DLK-1 expression in the head region of L4 animals of indicated genotypes. Cilia regions and amphid soma are indicated by white dashed boxes and enlarged in the left and right panels. Null *(0)* alleles used are *cebp-1(tm2807)*, *bbs-2(ok3035)*, *bbs-8(nx77)*, *osm-12(ju1748)*, and *mapk-15(ju1734)*. Scale bar: 5 μm. b) Quantification of GFP::DLK-1 expression in the cilia region of L4 animals of indicated genotypes. Null *(0)* alleles used are *cebp-1(tm2807)*, *bbs-2(ok3035)*, *bbs-8(nx77)*, and *osm-12(ju1748)*. Scale bar: 5 μm. Data are represented as mean ± SEM, with each dot representing an individual animal; the total numbers of animals per genotype are shown below the columns. Statistics: Welch's ANOVA test with multiple comparison, corrected with the FDR method of Benjamini and Hochberg. *P*-values are shown above brackets between the compared columns. c) Representative confocal Z-stack images of CEBP-1::GFP(*st12290*) in L2 animals of ctrl (wildtype), *osm-12(ju1748)*, and *mapk-15(gk1234)* background. Dashed boxes label the head ganglia, enlarged in the right panel. Scale bar: 10 μm.

Bardet–Biedl syndrome (BBS) is a genetic disorder arising from defects in primary cilia (Beales *et al*. 1999). BBSome is a conserved complex of 8 proteins that regulate cilia formation and function (Nachury *et al*. 2007; Loktev *et al*. 2008). In *C. elegans*, several mutations in *bbs* genes increased DLK-1 expression in the soma of ASH sensory neurons (Zhang *et al*. 2022). In our screen, we identified *ju1748* to contain a nonsense mutation at Arg496 of OSM-12*/*BBS-7, one of the core components of BBSome (Blacque *et al*. 2004). We tested a null allele of *osm-12/bbs-7* and observed increased GFP::DLK-1 accumulation in the cilia area. Double mutants of *osm-12(0); cebp-1(0)* showed further increased misaccumulation of GFP::DLK-1 in cilia (Fig. 4a and b). To test if increased GFP::DLK-1 activated its downstream signaling, we examined CEBP-1::GFP expression. We found that *osm-12(0)* showed increased CEBP-1::GFP in many ciliated neurons (Fig. 4c), suggesting that OSM-12 likely participates in the feedback control of DLK-1 by CEBP-1 as reported for other *ift* genes (Sun and Jin 2023). BBSome proteins can function together or independently (Mukhopadhyay *et al*. 2008; Kaplan *et al*. 2012). We tested loss-of-function mutations in *bbs-8* and *bbs-2*. *bbs-8(0)* showed GFP::DLK-1 misaccumulation in the cilia area and soma, which was enhanced by *cebp-1(0*). Although we noticed detectable difference in GFP::DLK-1 in *bbs-2(0)* single mutants, compared to the control, the extent of GFP::DLK-1 misaccumulation was unaffected by *cebp-1(0)* (Fig. 4a). This analysis suggests that components in BBSome may play different roles in regulating DLK-1 abundance, consistent with previous findings on BBSsome genes in other ciliary neuron function (Mukhopadhyay *et al*. 2008; Kaplan *et al*. 2012; Tian *et al*. 2023).

### MAPK-15 constrains DLK-1 abundance in a subset of ciliated neurons

MAPK-15 is a member of the atypical mitogen-activated protein kinase ERK7/8, and is expressed in many sensory neurons including URX, male-specific ciliated sensory neurons and other neurons (Bermingham *et al*. 2017; Kazatskaya *et al*. 2017; Piasecki *et al*. 2017). Loss of function in *mapk-15* alters cilia morphology and IFT transport in multiple sensory neurons (Bermingham *et al*. 2017; Kazatskaya *et al*. 2017). We found that *ju1734* contains a G-A nucleotide transition at the splicing donor site of exon-intron 4 junction of *mapk-15* (Table 1, Supplementary Fig. 1), likely causing out-of-frame after Ala147 in the kinase domain. *mapk-15(gk1234)*, a genetic null allele (Kazatskaya *et al*. 2017), also showed increased GFP::DLK-1 misaccumulation in the cilia region (Fig. 4a). Like *mapk-15(gk1234)*, *mapk-15(ju1734)* animals were defective in DiI uptake (Table 1). In the head ganglia, GFP::DLK-1 in *mapk-15(gk1234); cebp-1(0)* was visibly detected in the soma of about 10 sensory neurons (Fig. 4a). Consistently, we observed increased CEBP-1::GFP in the nuclei of about 10 sensory neurons in *mapk-15(0)* (Fig. 4c). GFP::DLK-1 intensity and pattern in the nerve ring, dorsal and ventral nerve cords were not visibly altered in either *mapk-15(gk1234)* or *mapk-15(ju1734)*, compared to controls. These results indicate that MAPK-15 constrains DLK-1 and CEBP-1 levels in select ciliated neurons whose identity remains to be determined.

### A novel mutation in HSP-90 regulates DLK-1 in ciliated neurons

**Fig. 5.**
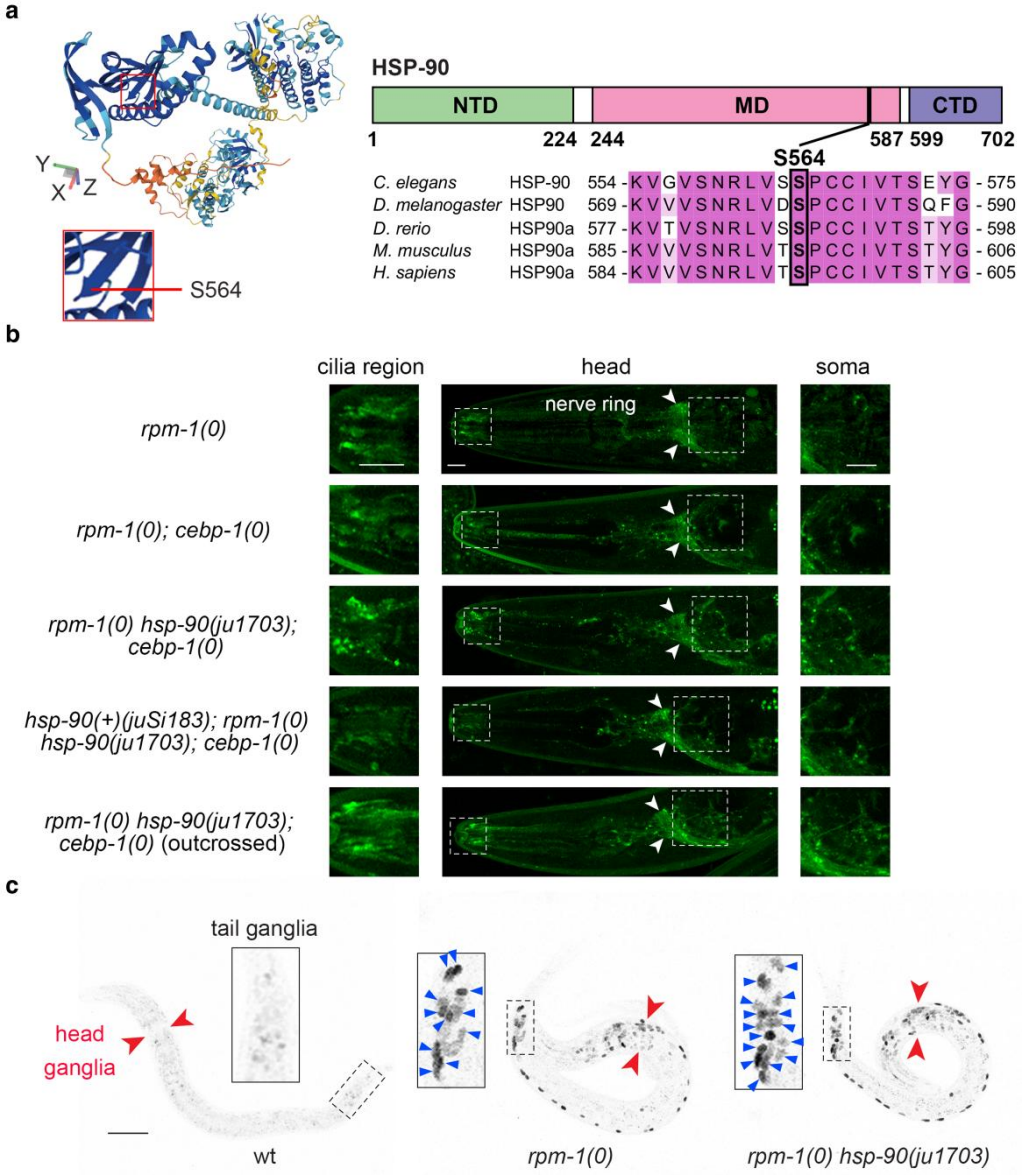
A unique mutation in HSP-90 regulates the abundance of GFP::DLK-1 in ciliated neurons. a) Predicted structure of HSP-90 by AlphaFold and alignment of HSP90 orthologs including *C. elegans* (Q18688), *Drosophila* (P02828), zebrafish (Q90474), mice (P07901), and human (P07900) in the amino acid sequence flanking Ser564 in *C. elegans*  HSP-90. b) Representative confocal Z-stack images of GFP::DLK-1 expression in the head region of L4 animals of indicated genotypes. Cilia regions and amphid soma are indicated by dashed boxes; nerve rings are pointed by arrowheads; cilia regions and amphid soma are enlarged in the left and right panels. Alleles used are *rpm-1(ju44); cebp-1(tm2807)*, *hsp-90(ju1703)*, and *juSi183[daf-21(+)]*. Scale bar: 5 μm. c) Representative confocal Z-stack images of CEBP-1::GFP(*st12290*) in L2 animals of ctrl (wildtype), *rpm-1(ju44)*, and *rpm-1(ju44) hsp-90(ju1703)* background. Red arrowheads point at the head ganglia, dashed boxes outline the tail ganglia, and blue arrowheads point at the soma in the tail ganglia that showed CEBP-1::GFP increase compared to ctrl. Scale bar: 10 μm.

Molecular chaperones regulate protein folding and play versatile roles in protein kinase mediated signal transduction (Saibil 2013). Previous studies have shown that Hsp70 and Hsp90 chaperone network is important for DLK protein stability in cultured mouse sensory neurons, in *Drosophila* neuromuscular junction, and in *C. elegans* mechanosensory neurons (Daviau *et al*. 2006; Karney-Grobe *et al*. 2018; Zheng *et al*. 2020). We found that *ju1703* mutation showed linkage to chromosome V and had a C-T nucleotide change in *hsp-90/daf-21*, resulting in Serine564 to Phenylalanine change in a conserved region among HSP90 proteins (Fig. 5a). We performed extensive outcrossing and eliminated many mutagenesis-induced SNPs on the right arm of chromosome V. We re-constructed a new strain (CZ30617 *GFP::dlk-1(ju1579)*, *rpm-1(0) hsp-90(ju1703)*, *cebp-1(0)*) and observed GFP::DLK-1 misaccumulation in cilia to a similar degree as the original isolate (Fig. 5b). Null mutants of *hsp-90* (*nr2081* and *ok1333*) arrest at young larval (L2) stages (Birnby *et al*. 2000). However, *rpm-1(0) hsp-90(ju1703)* animals were normal in development, locomotion and fertility, resembling *rpm-1(0)*. To confirm if *hsp-90(ju1703)* was causative to DLK-1 misaccumulation, we made a single-copy transgene expressing the full-length genomic DNA of *hsp-90*, *juSi183[daf-21(+)]*, which rescued the larval lethality of *hsp-90(nr2081)* (Supplementary Table 1). We then introduced *juSi183* to *GFP::dlk-1(ju1579)*, *rpm-1(0) hsp-90(ju1703); cebp-1(0)* animals and observed that GFP::DLK-1 abundance in cilia area was restored to the same level as in *rpm-1(0); cebp-1(0)* (Fig. 5b). These data support the conclusion that the missense S564F mutation in *hsp-90(ju1703)* causes selective increase of GFP::DLK-1 in sensory neurons. HSP-90 is ubiquitously expressed in all cell types (Wang et al. 2013). However, we observed increased GFP::DLK-1 only in the cilia region, compared to *rpm-1(0); cebp-1(0)* control. Moreover, in *rpm-1(0) hsp-90(ju1703)* animals, CEBP-1::GFP was visibly increased in some neurons in the head and tail ganglia, comparing to *rpm-1(0)* control (Fig. 5c). These results suggest that *hsp-90(S564F)* likely alters, but not eliminates, the function of *hsp-90* in ciliated neurons.

### The ODR-1 guanylyl cyclase represses DLK-1 in AWB and AWC neurons

**Fig. 6.**
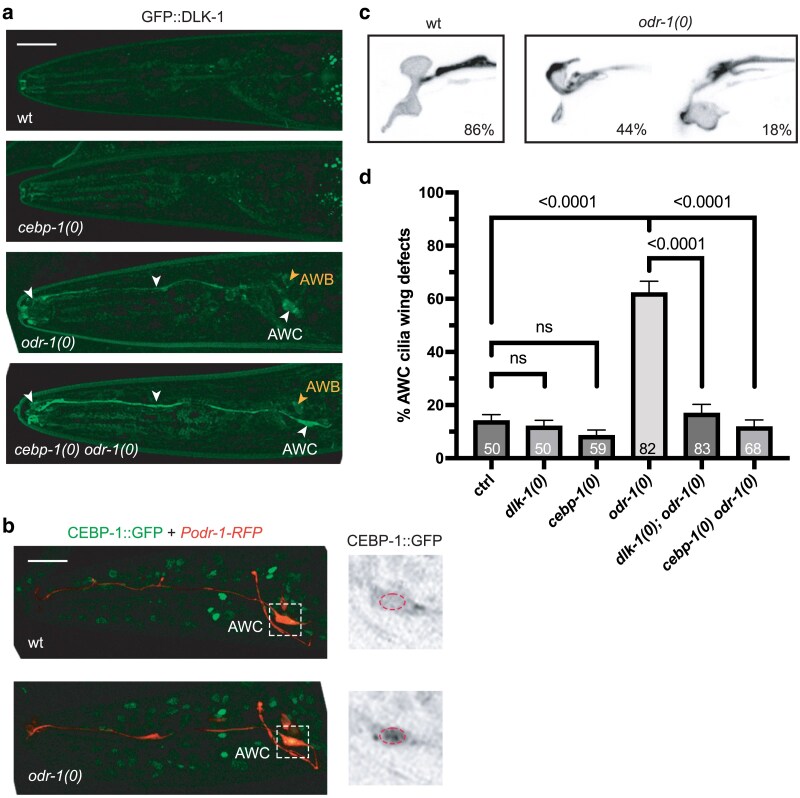
ODR-1 represses the abundance and signaling of DLK-1 and CEBP-1 in AWC neurons. a) Representative confocal Z-stack images of GFP::DLK-1 expression in the head region of L4 animals in wildtype (wt), *odr-1(n1936)*, and *cebp-1(tm2807) odr-1(n1936)* background. Cilia, dendrites and soma of AWC are indicated by white arrowheads; AWB soma are indicated by yellow arrowheads. Scale bar: 20 μm. b) Representative confocal Z-stack images of co-expressed CEBP-1::GFP*(st12290)* and *Podr-1-RFP(oyIs44)* in the head ganglia of L4 animals in wildtype (wt) and *odr-1(n1936)* background. Dashes indicate AWC soma. Red dashed circles outline CEBP-1 signal in the nuclei of AWC. Scale bar: 20 μm. c) Representative confocal Z-stack images of AWC cilia morphology in wildtype (wt) and *odr-1(n1936)*. Percentages of each category among the population are labeled. d) Quantification of the percentage of AWC cilia morphology defects using *Podr-1-GFP(oyIs45)*. Data are represented as mean ± SEM, with the total number of animals per genotype shown within the columns. Statistics: Fisher exact test. *P*-values are shown above brackets between the compared columns.

The guanylyl cyclase ODR-1 has a key role in AWB and AWC mediated olfaction (Bargmann *et al*. 1993; L’Etoile & Bargmann 2000). From our genetic screen, we found 2 mutations, *ju1739* and *ju1705*, that showed elevated GFP::DLK-1 in AWB and AWC neurons, determined using *Podr-1-RFP(oyIs44)*. GFP::DLK-1 were evenly increased in AWB and AWC cilia, dendrites, and soma, but not in axons (Fig. 6a). *ju1739* contains a nonsense mutation at Trp202 in ODR-1, and *ju1705* contains a Ser894Phe missense mutation in the guanylyl cyclase domain (Supplementary Figs. 1 and 2b). We constructed trans-heterozygous animals of *ju1705/ju1739* and observed misaccumulation of GFP::DLK-1 indistinguishable from the homozygous animals of either *ju1705* or *ju1739*. We also tested an independent null allele, *odr-1(n1936)*, and observed increased GFP::DLK-1 in AWB and AWC, comparable to either *ju1705* or *ju1739* (Supplementary Fig. 1). We conclude that both *ju1705* and *ju1739* are loss-of-function mutations in *odr-1*. Compared to single mutants of *odr-1*(*n1936*, *ju1705* or *ju1739*), *cebp-1(0) odr-1(0)* double mutant exhibited further increased DLK-1 levels in AWC but not AWB neurons (Fig. 6a). Consistently, we observed that CEBP-1::GFP intensity was visibly increased in AWC neurons of *odr-1(0)*, compared to control animals (Fig. 6b). These findings reveal that DLK-1 expression and signaling activity in AWC neurons are sensitive to ODR-1-dependent regulation.

### DLK-1 to CEBP-1 signaling antagonizes ODR-1 in AWC cilia morphology

AWC cilia display 2 flattened membrane structures (Perkins *et al*. 1986). We found that in *odr-1* single mutants (*n1936*, *ju1705* or *ju1739*) AWC cilia showed variable defects, including reduced or thin wing cilia, absence of 1 cilia wing, stub-like structures at the proximate PCMC region, as well as the widened appearance of dendrites (Fig. 6c). Loss of function in *dlk-1* or *cebp-1* does not affect AWC cilia (Sun and Jin 2023). However, AWC cilia morphological defects of *odr-1* mutants were significantly reduced in the *dlk-1(0); odr-1(0)* and *cebp-1(0) odr-1(0)* double mutants (Fig. 6d). These results suggest that *odr-1* loss of function causes activation of DLK-1 to CEBP-1 signaling, which contributes to distorted AWC cilia morphology.

## Conclusions

Our visual genetic screen using GFP::DLK-1 expressed from the endogenous locus has revealed multiple regulators that constrain the abundance and activity of DLK-1 in ciliated sensory neurons. The analysis of the new mutations extends from our previous findings of intraflagellar mutants (Sun & Jin 2023) and reveals additional ciliated proteins that act in concert with CEBP-1 to keep DLK-1 expression levels at check. Our co-labeling studies of GFP::DLK-1 with the markers for amphid sheath cells and EVs, along with analysis of CEBP-1::GFP, suggest that GFP::DLK-1 is unlikely expressed in glial cells or released via EVs. While we did not test all components of *C. elegans* BBSome, our data are consistent with the previous report that BBSsomes repress DLK-1 expression in ciliated neurons (Zhang *et al*. 2022), although individual components of BBSome complex appear to modulate DLK-1 levels in different manners. We find that MAPK-15 represses DLK-1 levels in select ciliated neurons, consistent with the cell-specific expression of MAPK-15 (Bermingham *et al*. 2017; Kazatskaya *et al*. 2017; Piasecki *et al*. 2017), although the identity of these neurons remains to be determined. One unexpected finding is that the S564P mutation in the ubiquitously expressed chaperone HSP-90 causes selective misaccumulation of DLK-1 in ciliated neurons. HSP-90 is known to regulate distinct chemosensory responses (Birnby *et al*. 2000). Ser564 is highly conserved among all HSP90 proteins. It is conceivable that Ser564 may be phosphorylated in cell-type specific manner, which may play a role in regulating HSP90 interaction with its clients, such as DLK-1. Biochemical studies will be necessary to determine the mechanistic interaction between HSP-90 and DLK-1. Our data on the ODR-1 interaction with DLK-1 further extend neuron-type specific role of DLK-1. *odr-1* is known to function in multiple chemosensory responses. Unlike the other genes that are broadly involved in cilia transport and development, *odr-1* is expressed in a few sensory neurons and is not known to be involved in IFT transport. Our observation that AWC cilia are distorted in *odr-1* mutants is consistent with previous report that AWB cilia remodeling is dependent on *odr-1* (Mukhopadhyay *et al*. 2008). Loss of function in *odr-1* elevates DLK-1 and CEBP-1 levels primarily in AWC neurons. Removal of *dlk-1* or *cebp-1* rescued AWC cilia defects in *odr-1* mutants, suggesting a detrimental effect of activating DLK-1 signaling. It would be of future interest to determine the signaling network underlying ODR-1 and DLK-1 interaction. Taken together, our findings from the forward genetic screen reveal novel factors and pathways that constrain DLK-1 signaling in ciliated sensory neurons with sophisticated cell-type specificity.

## Supplementary Material

jkaf004_Supplementary_Data

## Data Availability

Information of new alleles will be curated in WormBase. Some strains containing new alleles will be deposited to CGC, and others strains will be available upon request. Supplemental material available at G3 online.
