# Supplementary material for: Multiple regulators constrain the abundance of Caenorhabditis elegans DLK-1 in ciliated sensory neurons
Source: G3 (Bethesda). 2025 Jan 24;15(3):jkaf004. doi: 10.1093/g3journal/jkaf004 (PMC11917482; doi:10.1093/g3journal/jkaf004)
Supplement: jkaf004_Supplementary_Data [file jkaf004_supplementary_data.pdf]

Multiple regulators constrain the abundance of *C. elegans* DLK-1 in ciliated sensory neurons

Yue Sun[1], Junxiang Zhou[1,#], Arunima Debnath[1,#], Bokun Xie[1,#], Zhiping Wang[1], Yishi Jin[1,2,*]

Supplemental Materials

2 Figures and 2 Tables

Supplemental Figure legends

Figure S1. Illustrations of genes and mutations that cause DLK-1 misaccumulation in ciliated sensory neurons.

Predicted exon/intron structure of *ifta-1, dyf-3, dyf-11, che-2, che-3, osm-5, osm-12, mks-5, dyf-5, hsp-90, mapk-5, odr-1* in the genomic locus, adapted from illustrations in WormBase. The mutations identified in the screen that caused DLK-1 accumulation in ciliated neurons are indicated by arrowheads, along with predicted amino acid change.

Figure S2. Predicted protein structures of DYF-5 and ODR-1.

A. AlphaFold predicted structure of DYF-5(B3WFY8). Kinase domain is outlined in a black box; the mutated Lys6 is indicated by red line. **B**. AlphaFold predicted structure of ODR-1(B1Q257). The guanyl cyclase (GC) domain is outlined in a black box; the mutated Ser894 is indicated by red line

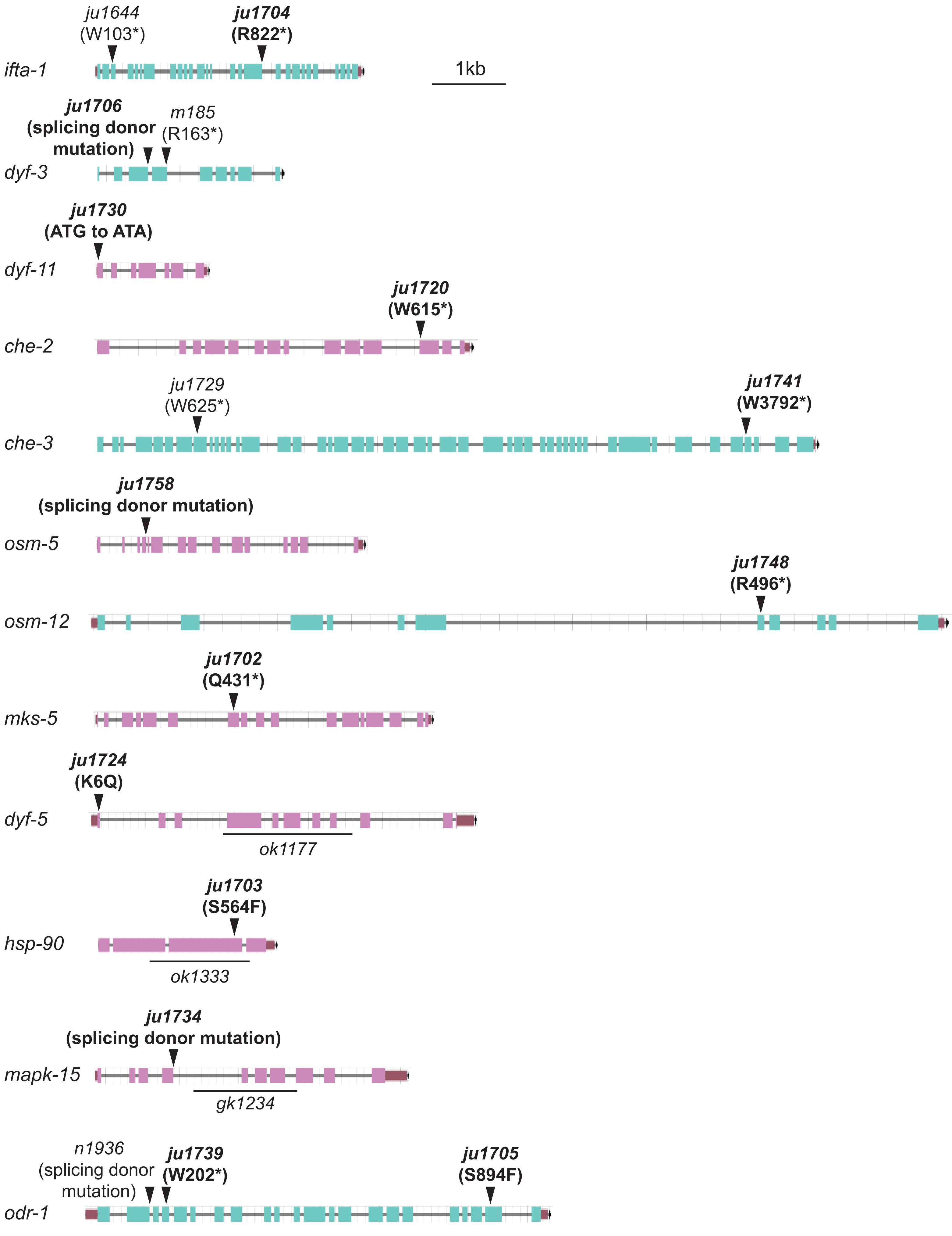

ju1644
(W103*)
ju1704
(R822*)
ifta-1
1kb
ju1706
(splicing donor
mutation)
m185
(R163*)
dyf-3
ju1730
(ATG to ATA)
dyf-11
ju1720
(W615*)
che-2
ju1729
(W625*)
ju1741
(W3792*)
che-3
ju1758
(splicing donor mutation)
osm-5
ju1748
(R496*)
osm-12
ju1702
(Q431*)
mks-5
ju1724
(K6Q)
dyf-5
ok1177
ju1703
(S564F)
hsp-90
ok1333
ju1734
(splicing donor mutation)
mapk-15
gk1234
n1936
(splicing donor
mutation)
ju1739
(W202*)
ju1705
(S894F)
odr-1

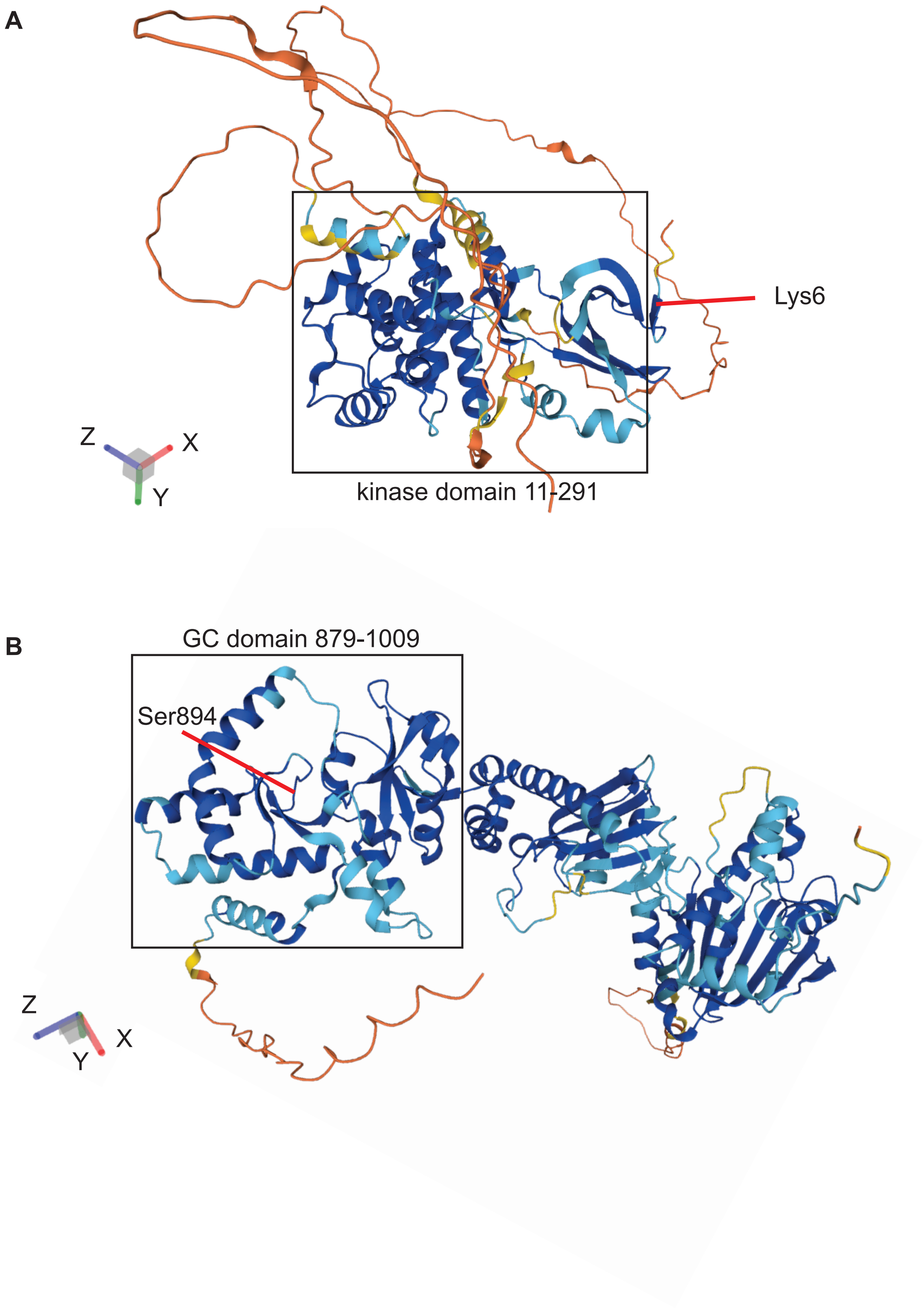
A
Lys6
kinase domain 11-291
Z
X
Y
B
GC domain 879-1009
Ser894
Z
X
Y

Table S1. Strain list

Strain and genotype	Relevant Figures, Table 1 and use
CZ25941 *GFP::dlk-1(ju1579) I*	Fig 1-7 and Table 1, control
CZ27822 *GFP::dlk-1(ju1579) I; cebp-1(tm2807) X*	Fig 1-7 and Table 1, control
CZ27137 *GFP::dlk-1(ju1579) I; ifta-1(ju1644) X*	Fig 1, control
CZ27185 *GFP::dlk-1(ju1579) I; cebp-1(tm2807) ifta-1(ju1644) X*	Fig 1, Table 1, control
CZ26350 *GFP::dlk-1(ju1579) I; rpm-1(ju44) V; cebp-1(tm2807) X*	Fig 1, Table 1, control
CZ27245 *GFP::dlk-1(ju1579) I; rpm-1(ju44) V; cebp-1(tm2807) odr-1(ju1705) X*	non-outcrossed mutant, WGS
CZ27246 *GFP::dlk-1(ju1579) I; dyf-3(ju1706) IV; rpm-1(ju44) V; cebp-1(tm2807) X*	non-outcrossed mutant, WGS
CZ27260 *GFP::dlk-1(ju1579) I; rpm-1(ju44) V; cebp-1(tm2807) che-2(ju1720) X*	non-outcrossed mutant, WGS
CZ27274 *GFP::dlk-1(ju1579) I; mapk-15(ju1734) III; rpm-1(ju44) V; cebp-1(tm2807) X*	non-outcrossed mutant, WGS
CZ27279 *GFP::dlk-1(ju1579) I; rpm-1(ju44) V; cebp-1(tm2807) odr-1(ju1739) X*	non-outcrossed mutant, WGS
CZ27288 *GFP::dlk-1(ju1579) I; osm-12(ju1748) III; rpm-1(ju44) V; cebp-1(tm2807) X*	non-outcrossed mutant, WGS
CZ26468 *GFP::dlk-1(ju1579) I; rpm-1(ju44) V; cebp-1(tm2807) X; ju1645*	non-outcrossed mutant
CZ27242 *GFP::dlk-1(ju1579) I; mks-5(ju1702) II; rpm-1(ju44) V; cebp-1(tm2807) X;*	non-outcrossed mutant, WGS
CZ27243 *GFP::dlk-1(ju1579) I; rpm-1(ju44) hsp-90(ju1703) V; cebp-1(tm2807) X;*	non-outcrossed mutant, WGS
CZ27244 *GFP::dlk-1(ju1579) I; rpm-1(ju44) V; cebp-1(tm2807) ifta-1(ju1704) X;*	non-outcrossed mutant
CZ27250 *GFP::dlk-1(ju1579) I; rpm-1(ju44) V; cebp-1(tm2807) X; ju1710*	non-outcrossed mutant, WGS
CZ27264 *dyf-5(ju1724) GFP::dlk-1(ju1579) I; rpm-1(ju44) V; cebp-1(tm2807) X*	non-outcrossed mutant, WGS
CZ27270 *GFP::dlk-1(ju1579) I; rpm-1(ju44) V; cebp-1(tm2807) dyf-11(ju1730) X*	non-outcrossed mutant, WGS
CZ27276 *GFP::dlk-1(ju1579) I; rpm-1(ju44) V; cebp-1(tm2807) X; ju1736*	non-outcrossed mutant, WGS
CZ27281 *GFP::dlk-1(ju1579) I; rpm-1(ju44) V; cebp-1(tm2807) X; ju1741*	non-outcrossed mutant, WGS
CZ27282 *che-3(ju1742) GFP::dlk-1(ju1579) I; rpm-1(ju44) V; cebp-1(tm2807) X*	non-outcrossed mutant, WGS
CZ27285 *GFP::dlk-1(ju1579) I; rpm-1(ju44) V; cebp-1(tm2807) X; ju1745*	non-outcrossed mutant
CZ27290 *GFP::dlk-1(ju1579) I; rpm-1(ju44) V; cebp-1(tm2807) X; ju1750*	non-outcrossed mutant, WGS
CZ27293 *GFP::dlk-1(ju1579) I; rpm-1(ju44) V; cebp-1(tm2807) X; ju1753*	non-outcrossed mutant, WGS
CZ27298 *GFP::dlk-1(ju1579) I; rpm-1(ju44) V; cebp-1(tm2807) osm-5(ju1758) X*	non-outcrossed mutant

CZ27002 *GFP::dlk-1(ju1579) I; cebp-1(tm2807) X; ju1645*	WGS
CZ27014 *GFP::dlk-1(ju1579) I; cebp-1(tm2807) X; ju1645*	Fig 1, Table 1, WGS
CZ30395 *GFP::dlk-1(ju1579) I; cebp-1(tm2807) odr-1(ju1705) X*	Fig 1, Table 1
CZ30396 *GFP::dlk-1(ju1579) I; cebp-1(tm2807) odr-1(ju1739) X*	Fig 1, Table 1
CZ30397 *GFP::dlk-1(ju1579) I; rpm-1(ju44) daf-21(ju1703) V cebp-1(tm2807) X*	Fig 1, Table 1
CZ30398 *dyf-5(ju1724) GFP::dlk-1(ju1579) I; cebp-1(tm2807) X*	Fig 1, Table 1
CZ30399 *che-3(ju1742) GFP::dlk-1(ju1579) I; cebp-1(tm2807) X*	Fig 1, Table 1, WGS
CZ30400 *GFP::dlk-1(ju1579) I; cebp-1(tm2807) X; ju1745*	Fig 1, Table 1
CZ30401 *GFP::dlk-1(ju1579) I; cebp-1(tm2807) X; ju1750*	Fig 1, Table 1, WGS
CZ30402 *GFP::dlk-1(ju1579) I; cebp-1(tm2807) X; ju1753*	Fig 1, Table 1, WGS
CZ30403 *GFP::dlk-1(ju1579) I; mks-5(ju1702) II; cebp-1(tm2807) X*	Fig 1, Table 1, WGS
CZ30404 *GFP::dlk-1(ju1579) I; cebp-1(tm2807) ifta-1(ju1704) X*	Fig 1, Table 1, WGS
CZ30405 *GFP::dlk-1(ju1579) I; rpm-1(ju44) V cebp-1(tm2807) X; ju1710*	Fig 1
CZ30495 *GFP::dlk-1(ju1579) I; rpm-1(ju44) V cebp-1(tm2807) X; ju1710*	Table 1, WGS
CZ30406 *GFP::dlk-1(ju1579) I; cebp-1(tm2807) X; ju1741*	Fig 1, Table 1, WGS
CZ30407 *GFP::dlk-1(ju1579) I; cebp-1(tm2807) osm-5(ju1758) X*	Fig 1, Table 1, WGS
CZ30408 *GFP::dlk-1(ju1579) I; cebp-1(tm2807) X; ju1736*	Fig 1, Table 1, WGS
CZ28087 *GFP::dlk-1(ju1579) I; cebp-1(tm2807) dyf-11(ju1730) X*	Fig 1, Table 1, WGS
CZ30236 *GFP::dlk-1(ju1579) I; mapk-15(ju1734) III; cebp-1(tm2807) X*	Fig 1, Table 1
CZ27716 *GFP::dlk-1(ju1579) I; dyf-3(m185) IV*	Fig 2, supporting evidence that *dyf-3* regulates DLK-1
CZ30232 *GFP::dlk-1(ju1579) I; dyf-3(ju1706) IV*	Fig 2
CZ30233 *GFP::dlk-1(ju1579) I; dyf-3(ju1706) IV; cebp-1(tm2807) X*	Fig 1,2, Table 1
CZ30234 *GFP::dlk-1(ju1579) I; cebp-1(tm2807) che-2(ju1720) X*	Fig 1,2, Table 1
CZ29852 *GFP::dlk-1(ju1579) I; osm-3(p802) IV*	Fig 2
CZ29854 *GFP::dlk-1(ju1579) I; kap-1(ok676) II*	Fig 2
CZ30630 *GFP::dlk-1(ju1579) I; kap-1(ok676) II; osm-3(p802) IV*	Fig 2
OS2042 *Podr-1::YFP(oyIs45) V; nsEx1153[F16F9.3p-mCherry + itr-1P-CFP]*	Fig 3, reporter control
CZ30611 *GFP::dlk-1(ju1579) I; cebp-1(tm2807) ifta-1(ju1644) X; nsEx1153[F16F9.3p-mCherry + itr-1P-CFP]*	Fig 3
OQ366 *tsp-6::wrmScarlet(syb4122) X ; PF16F9.3::CFP(juEx8158)*	Fig 3, reporter control
CZ30576 *GFP::dlk-1(ju1579) I; cebp-1(tm2807) ifta-1(ju1644) X; PF16F9.3::CFP(juEx8158)*	Fig 3
CZ30612 *GFP::dlk-1(ju1579) che-3(ju1729) I; tsp-6::wrmScarlet(syb4122) X*	Fig 3
CZ30238 *GFP::dlk-1(ju1579) I; osm-12(ju1748) III; cebp-1(tm2807) X*	Fig 1,4, Table 1
CZ30237 *GFP::dlk-1(ju1579) I; osm-12(ju1748) III*	Fig 4
CZ30605 *GFP::dlk-1(ju1579) I; bbs-2(ok3035) IV*	Fig 4
CZ30606 *GFP::dlk-1(ju1579) I; bbs-2(ok3035) IV; cebp-1(tm2807) X*	Fig 4
CZ30607 *GFP::dlk-1(ju1579) I; bbs-8(nx77) V*	Fig 4
CZ30608 *GFP::dlk-1(ju1579) I; bbs-8(nx77) V; cebp-1(tm2807) X*	Fig 4

CZ30613 *osm-12(ju1748) III; cebp-1::GFP(st12290) X*	Fig 4
CZ28270 *GFP::dlk-1(ju1579) I; mapk-15(gk1234) III*	Fig 4
CZ28271 *GFP::dlk-1(ju1579) I; mapk-15(gk1234) III; cebp-1(tm2807) X*	Fig 4
CZ30397 *GFP::dlk-1(ju1579) I; rpm-1(ju44) daf-21(ju1703) V, cebp-1(tm2807) X*	Fig 5
CZ20899 *mec-4-GFP(zdIs5) I; juSi183[LoxP-Pdaf-21-daf-21-LoxP] II; daf-21(nr2081) V*	Fig 5, supporting evidence that *juSi183* is fully functional
CZ30577 *GFP::dlk-1(ju1579) I; juSi183[LoxP-Pdaf-21-daf-21-LoxP] II; rpm-1(ju44) daf-21(ju1703) V*	Fig 5
CZ30578 *GFP::dlk-1(ju1579) I; juSi183[LoxP-Pdaf-21-daf-21-LoxP] II; rpm-1(ju44) daf-21(ju1703) V; cebp-1(tm2807) X*	Fig 5
CZ30579 *rpm-1(ju44) daf-21(ju1703) V*	Fig 5
CZ30617 *GFP::dlk-1(ju1579) I; rpm-1(ju44) daf-21(ju1703) V, cebp-1(tm2807) X*	Fig 5
CZ30629 *rpm-1(ju44) daf-21(ju1703) V; cebp-1::GFP(st12290) X*	Fig 5
CZ28894 *rpm-1(ju44ts) V; cebp-1::GFP(st12290) X*	Fig 5
CZ28272 *GFP::dlk-1(ju1579) I; odr-1(n1936) X*	Fig 6
CZ28273 *GFP::dlk-1(ju1579) I; cebp-1(tm2807) odr-1(n1936) X*	Fig 6
CZ28578 *[Podr-1-RFP + lin-15(+)](oyIs44) V; cebp-1::TY1::EGFP::3xFLAG(st12290) X*	Fig 6
CZ28887 *[Podr-1-RFP + lin-15(+)](oyIs44) V; cebp-1::TY1::EGFP::3xFLAG(st12290) odr-1(n1936) X*	Fig 6
PY2417 *Podr-1::YFP(oyIs44) V*	Fig 6, reporter control
CZ29014 *Podr-1::RFP(oyIs44) V ; cebp-1(tm2807) X*	Fig 6
CZ30071 *Podr-1::RFP(oyIs44) V ; odr-1(n1936) X*	Fig 6
CZ30070 *Podr-1::RFP(oyIs44) V ; odr-1(ju1705) X*	Fig 6
CZ30069 *Podr-1::RFP(oyIs44) V ; odr-1(ju1739) X*	Fig 6
CZ29984 *Podr-1::RFP(oyIs44) V ; cebp-1(tm2807) X odr-1(n1936) X*	Fig 6
CZ29985 *Podr-1::RFP(oyIs44) V ; cebp-1(tm2807) X odr-1(ju1705) X*	Fig 6
CZ29987 *Podr-1::RFP(oyIs44) V ; cebp-1(tm2807) X odr-1(ju1739) X*	Fig 6
CZ30123 *Podr-1::RFP(oyIs44) V ; odr-1(ju1705) X ; ceh-36p3::odr-1(udEx307)*	Fig 6
CZ30125 *Podr-1::RFP(oyIs44) V ; odr-1(ju1739) X ; ceh-36p3::odr-1(udEx307)*	Fig 6
CZ30127 *Podr-1::RFP(oyIs44) V ; odr-1(n1936) X ; ceh-36p3::odr-1(udEx307)*	Fig 6
CZ29017 *Podr-1::RFP(oyIs44) V ; dlk-1(km12) I*	Fig 6
CZ30244 *Podr-1::RFP(oyIs44) V ; dlk-1(tm4024) I*	Fig 6
CZ30200 *Podr-1::RFP(oyIs44) V ; dlk-1(km12) I ; odr-1(n1936) X*	Fig 6
CZ30245 *Podr-1::RFP(oyIs44) V ; dlk-1(tm4024) I ; odr-1(n1936) X*	Fig 6
CZ30444 *Podr-1::YFP(oyIs45) V*	Fig 6, reporter control
CZ30517 *Podr-1::YFP(oyIs45) V ; odr-1(ju1739) X*	Fig 6
CZ30518 *Podr-1::YFP(oyIs45) V ; cebp-1(tm2807) X*	Fig 6
CZ30519 *Podr-1::YFP(oyIs45) V ; cebp-1(tm2807) X odr-1(ju1739) X*	Fig 6
CZ30520 *Podr-1::YFP(oyIs45) V ; dlk-1(km12) I*	Fig 6
CZ30521 *Podr-1::YFP(oyIs45) V ; dlk-1(km12) I ; odr-1(n1936) X*	Fig 6
CZ9367 *Pstr-1::GFP (kyIs104) X*	Fig 6, reporter control
CZ30570 *Pstr-1::GFP (kyIs104) odr-1 (n1936) X*	Fig 6

CZ30571 *Pstr-1::GFP (kyIs104) odr-1 (ju1739) X*	Fig 6
CZ30572 *dlk-1 (km12) I; Pstr-1::GFP (kyIs104) X*	Fig 6
CZ30573 *dlk-1 (km12) I; Pstr-1::GFP (kyIs104) odr-1 (n1936) X*	Fig 6

Table S2. Alleles and genotyping primers

Allele	Gene	Mutation	Primer	Primer sequence	PCR product size
ju1702	*mks-5*	WT - gatcttctt**C**aaaaattata *ju1702* - gatcttctt**T**aaaaattata	YJ12878	GTCGAATGCGAAGAGCTGACG	429 bp
			YJ12879	CAGGCTGTTGTCCTGAATATGC	
ju1703	*hsp-90*	WT - gtcttgtct**C**ttccccatgc *ju1703* - gtcttgtct**T**ttccccatgc	YJ12872	CATGGAGAACTGCGAAGAGC	775 bp
			YJ12873	GGCCATGTATCCCATAGTAGA GG	
ju1704	*ifta-1*	WT - gctgataga**C**ggcaagtcaa *ju1704* - gctgataga**T**ggcaagtcaa	YJ12880	GAAGCCATAGATTACGCGGAG C	465 bp
			YJ12881	GAGTAACTGATTTCGTGGTCCG	
ju1705	*odr-1*	WT - atggttgctt**C**tggtgtccc *ju1705*- atggttgctt**T**tggtgtccc	YJ12860	CACTGTAATCAAGAGACACGA TGC	358 bp
			YJ12861	GTCGCCGAAGAGGCAATACC	
ju1706	*dyf-3*	WT - ctcgtcgaag**G**tgggtctca *ju1706* - ctcgtcgaag**A**tgggtctca	YJ12862	AATCCAACCTTGACGCACACG	724 bp
			YJ12863	CTAGTGGTATATGCAGGGCTTC AAG	
ju1720	*che-2*	WT - cacaatgtg**G**gcgacttttg *ju1720* - cacaatgtg**A**gcgacttttg	YJ12864	ACGTGTTGCTATAAATGACC	279 bp
			YJ12865	ATATTCAACATAAGCGAGCGA AAGC	
ju1724	*dyf-5*	WT - tcggctgtt**A**aacttgctga *ju1724* - tcggctgtt**C**aacttgctga	YJ12888	CCGCCGTTTGCTCTTGGTTAC	753 bp
			YJ12889	GCTCGACGGGCGCTAATATG	
ju1730	*dyf-11*	WT - aagtgcgat**G**agcgttgagg *ju1724* - aagtgcgat**A**agcgttgagg	YJ12882	CTGCTCTCATCGTCTCACGC	757 bp
			YJ12883	GGTTTCTTCCGCGTCCTTCC	
ju1734	*mapk-15*	WT - gttacaccg**G**tgagtacgag *ju1734* - gttacaccg**A**tgagtacgag	YJ12866	TTGAGTTCATGGAAGCTGATC	620 bp
			YJ12867	GTGACTGACACCGCTGATTGG	
ju1739	*odr-1*	WT - atgtggattg**G**acgaaggtt *ju1739* - atgtggattg**A**acgaaggtt	YJ12868	AGAGCTGGAACAAGTTGAGG	424 bp
			YJ12869	CCTGATAGTGATCGTATGCCA	
ju1741	*che-3*	WT - ggctgattg**G**gaatttgtga *ju1741* - ggctgattg**A**gaatttgtga	YJ12884	ACTTCCGTCTATGGCTCACC	676 bp
			YJ12885	CGGTAGTCCAAACAGATAAGG C	
ju1748	*osm-12*	WT - ctctcacatg**C**gatcacatg *ju1748* - ctctcacatg**T**gatcacatg	YJ12870	TCAAGGCTTTATCCTCTCACAT G	278 bp
			YJ12871	AACTTGAAGCTGTGTTCCTCC	
ju1758	*osm-5*	WT - caaataaag**G**tgacatccgc	YJ12886	GGCTAACAACAATGAACCTGC G	388 bp

		ju1758 - caaataaag**A**tgacatccgc	YJ12887	TCTACCAATCCCTGTTGCTCCC	
ju1579 (Sun & Jin, 2023)	*dlk-1*	1035 bp insertion	YJ11607	AAATCTAGGGCCTTACGAC	WT - 759 bp *ju1579* - 1794 bp
			YJ11608	CGTCGTCTCACTATTTGC	
km12 (Bounoutas et al., 2009)	*dlk-1*	734 bp deletion	YJ9794	GCTATCTCCGAACTTGAATG	WT - 1901 bp *km12* - 1167 bp
			YJ12644	TCTCTGTAAGAATCCCATGC	
ju44 (Zhen et al., 2000)	*rpm-1*	WT - tgtcttggg**C**agacttgaact *ju44* - tgtcttggg**T**agacttgaact	YJ2949	GGAAAGAAGATGGAATGGTC	(AccI digestion) WT - 759 bp *ju44* - 429 + 330 bp
			YJ9183	CAAATTCCACAACCCATTGC	
tm2807 (Yan et al., 2009)	*cebp-1*	479 bp deletion	YJ5442	CTGATAACCTTCTGGGTGTGTCC	WT - 1366 bp *tm2807* - 887 bp
			YJ5443	GCCGTGGAATAAATATGAGG	
m185 (Murayama et al., 2005)	*dyf-3*	WT – gagtgattc**G**gacctcctgc *m185* - gagtgattc**A**gacctcctgc	YJ12856	AATCCAACCTTGACGCACACG	724 bp
			YJ12857	CTAGTGGTATATGCAGGGCTTCAAG	
p802 (Shakir et al., 1993)	*osm-3*	WT - tttcctcct**G**gtactctcga *p802* - tttcctcct**A**gtactctcga	YJ12844	TTTAGTCTCTGCCTCCTTCAGC	854 bp
			YJ12845	AAGCATATACCCTACAGGGACAG	
ok676 (Snow et al., 2004)	*kap-1*	989 bp deletion 3 bp insertion	YJ8611	AAGCTCGCTTGAGTGCTTTT	WT - 1393 bp *ok676* - 407 bp
			YJ8612	TTGATTGACCCAACTCACCA	
ok3035 (CGC)	*bbs-2*	About 400 bp deletion	SD21071	GACCTCAGTTGGATGATAATGTCG	WT - 1272 bp *ok3035* - about 872 bp
			SD21072	GTGGATCAACGTTGCCATCA	
nx77 (Blacque et al., 2004)	*bbs-8*	819 bp deletion	SD21073	ATGAGTGGCGAATCAGTTATCGAG	WT - 1728 bp *nx77* - 909 bp
			SD21074	CTGAGTATAGACGAGAGCGC	
ok1351 (CGC)	*osm-12*	About 1300 bp deletion	YJ12846	TTCCACGTCACCAGATACCA	WT - 2849 bp *ok1351* - about 1549 bp
			YJ12847	CCCCACAGTGCTCCTACAAT	
gk1234 (Kazatskaya et al., 2017)	*mapk-15*	1376 bp deletion	YJ12858	GGTGAGTACGAGCAGTCTGG	WT - 2074 bp *gk1234* - 698 bp
			YJ12859	GGAGCACGACTCTTATCTTCGC	
n1936 (L'Etoile & Bargmann, 2000)	*odr-1*	WT - caagttgag**G**taattcaaaa *n1936* - caagttgag**A**taattcaaaa	YJ12681	AAATCAGCCTCCTACGCTATCAACG	508 bp
			YJ12582	GGTCAACCTTCGTCCAATCCAC	

References

Blacque, O. E., Reardon, M. J., Li, C., McCarthy, J., Mahjoub, M. R., Ansley, S. J., Badano, J. L., Mah, A. K., Beales, P. L., Davidson, W. S., Johnsen, R. C., Audeh, M., Plasterk, R. H., Baillie, D. L., Katsanis, N., Quarmby, L. M., Wicks, S. R., & Leroux, M. R. (2004). Loss of *C. elegans* BBS-7 and BBS-8 protein function results in cilia defects and compromised intraflagellar transport. *Genes & development*, *18*(13), 1630–1642.

Bounoutas, A., Zheng, Q., Nonet, M. L., & Chalfie, M. (2009). *mec-15* encodes an F-box protein required for touch receptor neuron mechanosensation, synapse formation and development. *Genetics*, *183*(2), 607–4SI.

Kazatskaya, A., Kuhns, S., Lambacher, N. J., Kennedy, J. E., Brear, A. G., McManus, G. J., Sengupta, P., & Blacque, O. E. (2017). Primary Cilium Formation and Ciliary Protein Trafficking Is Regulated by the Atypical MAP Kinase MAPK15 in *Caenorhabditis elegans* and Human Cells. *Genetics*, *207*(4), 1423–1440.

L'Etoile, N. D., & Bargmann, C. I. (2000). Olfaction and odor discrimination are mediated by the *C. elegans* guanylyl cyclase ODR-1. *Neuron*, *25*(3), 575–586.

Murayama, T., Toh, Y., Ohshima, Y., & Koga, M. (2005). The dyf-3 gene encodes a novel protein required for sensory cilium formation in *Caenorhabditis elegans*. *Journal of molecular biology*, *346*(3), 677–687.

Perkins, L. A., Hedgecock, E. M., Thomson, J. N., & Culotti, J. G. (1986). Mutant sensory cilia in the nematode *Caenorhabditis elegans*. *Developmental biology*, *117*(2), 456–487.

Sun, Y., & Jin, Y. (2023). An intraflagellar transport dependent negative feedback regulates the MAPKKK DLK-1 to protect cilia from degeneration. *Proceedings of the National Academy of Sciences*, *120*(39), e2302801120.

Shakir, M. A., Fukushige, T., Yasuda, H., Miwa, J., & Siddiqui, S. S. (1993). *C. elegans osm-3* gene mediating osmotic avoidance behaviour encodes a kinesin-like protein. *Neuroreport*, *4*(7), 891–894.

Snow, J. J., Ou, G., Gunnarson, A. L., Walker, M. R., Zhou, H. M., Brust-Mascher, I., & Scholey, J. M. (2004). Two anterograde intraflagellar transport motors cooperate to build sensory cilia on *C. elegans* neurons. *Nature cell biology*, *6*(11), 1109–1113.

Yan, D., Wu, Z., Chisholm, A. D., & Jin, Y. (2009). The DLK-1 kinase promotes mRNA stability and local translation in *C. elegans* synapses and axon regeneration. *Cell, 138*(5), 1005–1018.

Zhen, M., Huang, X., Bamber, B., & Jin, Y. (2000). Regulation of presynaptic terminal organization by *C. elegans* RPM-1, a putative guanine nucleotide exchanger with a RING-H2 finger domain. *Neuron*, *26*(2), 331–343.
